# Symmetry in Signals: A New Insight

**DOI:** 10.3390/e26110941

**Published:** 2024-11-02

**Authors:** Jean-Marc Girault

**Affiliations:** 1 Groupe ESEO, 49000 Angers, France; jean-marc.girault@eseo.fr; 2 Laboratoire d’Acoustique de l’Université du Mans (LAUM), UMR 6613, Institut d’Acoustique—Graduate School (IA-GS), CNRS, Le Mans Université, 72085 Le Mans, France

**Keywords:** symmetry, distortion, symmetry group, irreversibility, signal, symmetrometry, symmentropy, distorsymmetry, THD

## Abstract

Symmetry is a fundamental property of many natural systems, which is observable through signals. In most out-of-equilibrium complex dynamic systems, the observed signals are asymmetric. However, for certain operating modes, some systems have demonstrated a resurgence of symmetry in their signals. Research has naturally focused on examining time invariance to quantify this symmetry. Measures based on the statistical and harmonic properties of signals have been proposed, but most of them focused on harmonic distortion without explicitly measuring symmetry. This paper introduces a new mathematical framework based on group theory for analyzing signal symmetry beyond time invariance. It presents new indicators to evaluate different types of symmetry in non-stochastic symmetric signals. Both periodic and non-periodic symmetric signals are analyzed to formalize the problem. The study raises critical questions about the completeness of symmetry in signals and proposes a new classification for periodic and non-periodic signals that goes beyond the traditional classification based on Fourier coefficients. Furthermore, new measures such as “symmetrometry” and “distorsymmetry” are introduced to quantify symmetry. These measures outperform traditional indicators like Total Harmonic Distortion (THD) and provide a more accurate measurement of symmetry in complex signals from applications where duty cycle plays a major role.

## 1. Problem Statement

Symmetry is a fundamental property of the laws of nature, which is present in numerous dynamic systems, and it is often identified through observables such as signals [1,2,3]. To better understand these systems, researchers have sought to characterize them by evaluating the level of symmetry in their associated signals, as demonstrated in studies like [3,4,5,6,7,8,9,10,11]. This approach has been particularly relevant in the biomedical field, where signal symmetry is used to characterize the cardiovascular system [4,5,7]. For complex non-equilibrium biomedical systems, their temporal evolution is marked by the arrow of time, which annihilates any global symmetry in the signal, making it asymmetric. However, in specific pathologies like Cheyne–Stokes [12], an increase in symmetry has been observed [4,5], raising the question of how to quantify this gain in symmetry.

To address this issue, most researchers have focused on time irreversibility, studying invariance under time reversal [3,4,5,6,7]. Numerous metrics, each with varying degrees of effectiveness and based on statistical properties of signals, have been proposed. A recent review [8] reports nearly a dozen such metrics.

In fields such as telecommunications [13,14], audio electronics [15,16], electrical engineering [17,18], and ultrasonic medical imaging [19,20], there are currently no metrics available to quantify system performance based on symmetry properties. The only existing indicators measure harmonic distortion, including Total Harmonic Distortion (THD) [13,14] and the B/A coefficient in ultrasonic medical imaging [19,20]. In telecommunications, distortion of radio emissions helps evaluate interference effects during transmission. Similarly, Class C and D power amplifiers in audio electronics, though more efficient than traditional Class A, AB, and B amplifiers, introduce high harmonic content due to their non-linearity. However, these harmonic-based metrics are not explicitly linked to symmetry measurement.

Today, there is no mathematical framework that clearly and exhaustively describes all signal symmetries. For periodic signals, F-classification (see Appendix A.1) is the first framework. This classification uses the nullity of specific Fourier series coefficients to simplify calculations and classify signals based on symmetry properties. While this classification is valuable for periodic signals, it does not extend to non-periodic symmetric signals. For stochastic signals, symmetry is evaluated exclusively in terms of temporal irreversibility, and it is the invariance of statistical properties that is sought. Generally speaking, two approaches are envisaged for the study of stochastic signals. The first is to identify how time reversal modifies the statistical properties of the stochastic signal. The resulting tools seek to quantify which information is naturally oriented in the positive direction of time and which is in the opposite direction. The second approach links the intrinsic properties of the system to the symmetry of the time reversal. Breaking this symmetry leads to blatant asymmetry, resulting in significant dissipation quantifiable by a measure of entropy. For more details, see the article [8]. A complete classification of symmetric signals and a measure of symmetry levels is still needed.

In this article, we aim to build on previous approaches while introducing a new perspective on symmetric signal analysis. From a “signal” point of view, we propose an original mathematical framework, and based on this, we present new indicators that perform the following: (i) Account for symmetries beyond time reversal; (ii) Quantify the level of symmetry in non-stochastic signals. These new metrics are inspired by recent works [2,10].

**Figure 1:**
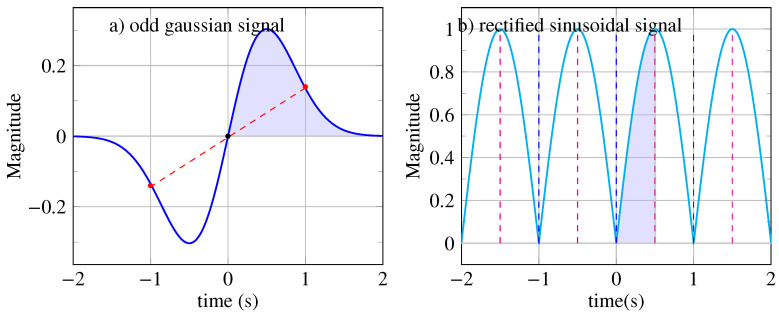
(**a**) Odd signal $x\left(t\right)=t\times \exp\left(-2t^{2}\right)$ with a single center of inversion at t=0. The Gaussian signal is obtained from a generating pattern whose area under the curve is colored in blue. (**b**) Periodic signal x(t)=|sin(2πt/T)| with a period T=1 s possessing symmetry axes at kT/2 (vertical dashed lines) where *k* is an integer. The rectified sinusoidal signal is obtained from a generating pattern whose area under the curve is colored in blue.

To clarify these concepts, let us examine two examples of symmetric signals. First, consider a finite-energy signal with unbounded support, $x\left(t\right)=t\times \exp\left(-2t^{2}\right)$. As shown in Figure 1a, there is a notable position at t=0, around which two equidistant points are opposed (marked by red points in Figure 1a). This is a center of inversion with an infinite range of symmetry (see Appendix A.2 for the definition of symmetry range). Thus, the signal exhibits odd symmetry, which can be confirmed by the following expression: $x\left(-t\right)=\left(-t\right)\times \exp(-2{\left(-t\right)}^{2})=-x\left(t\right)$.

*But if the signal’s mathematical form is unknown, how can we verify its odd symmetry? Furthermore, how can we measure the level of symmetry in the signal?*

Next, let us consider a signal with finite average power, x(t)=|sin(2πt/T)| with a period of T=1 s. Observing Figure 1b, we notice that at t=0, two equidistant points are equal, which indicates a mirror reflection axis with infinite symmetry range. This signal has even symmetry, x(t)=x(−t), as verified by the expression x(−t)=|sin(2π(−t)/T)|=x(t). Additionally, this mirror symmetry holds for all reflection axes at intervals of kT/2 (see Figure 1b). Over a time horizon of 4 s, we observe four patterns with a period of T=1 s and eight mirror axes.

*How can we account for all these mirror symmetries simultaneously and simply?*

At this stage, and to complement the previous inquiries, further questions are raised:

- *How can we verify the completeness of the symmetries present in a signal?*
- *What mathematical tools allow us to characterize a symmetric signal?*
- *Is there a framework that perfectly describes signal symmetry?*
- *Is there a more general classification of symmetrical signals that could encompass the F-classification?*
- *How many distinct types of periodic and non-periodic signals exist?*
- *Periodic signals have global symmetry with infinite range; what about signals with partial symmetry and limited local range?*
- *Are there metrics to measure the gain or loss of symmetry in a signal?*

In this article, we will address each of these questions, starting with the presentation of the chosen mathematical framework. We will then introduce various measures of symmetry levels and apply them to multiple examples. Finally, we conclude with a discussion and potential future directions.

## 2. Mathematical Framework

With the goal of providing clear answers to each of the previous questions, several concepts such as isometries, symmetry groups, generators, and classification will be addressed.

Let us begin by defining what a symmetric signal is. From a mathematical perspective, a signal x(t) is said to be symmetric if it is invariant under a transformation Γ[x(t)]=x(t). This transformation Γ[x(t)], which is an isometry, is detailed in the following section.

### 2.1. Isometries in Signal Analysis

An isometry is a geometric transformation that preserves the distances between two points; it does not distort either time or amplitude. An isometry is linear and satisfies the property Γ[x(t)+y(t),τ]=Γ[x(t),τ]+Γ[y(t),τ], where x(t) is the signal under study. For the study of signals, isometries can be summarized as translation, vertical reflection, inversion, and glide reflection. We will explore later why, among the set of possible isometries, only these four are considered. Additionally, these transformations can be composed, for example, as Γ[x(t),τ]∘Γ[x(t),−τ]=Γ[Γ[x(t),τ],−τ]=Γ[x(t),τ−τ]=Γ[x(t),0]=x(t).

Let us detail the four isometries:

1. The translation operation is defined by $x^{\prime }\left(t\right)=\Gamma_{T}\left[x\left(t\right),\tau \right]=x\left(t-\tau \right)$, where τ is a delay. The signal x(t) is invariant under translation if it satisfies the following: $x^{\prime }\left(t\right)=x\left(t\right)$. For τ=0, it follows that $\Gamma_{T}\left[x\left(t\right),0\right]=\Gamma_{\epsilon }\left[x\left(t\right)\right]=x\left(t\right)$ where $\Gamma_{\epsilon }\left[x\left(t\right)\right]=x\left(t\right)$ is the identity operation. The composition of translations results in $\Gamma_{T}\left[x\left(t\right),b\right]\;\circ \;\Gamma_{T}\left[x\left(t\right),a\right]=x\left(t-\left(b+a\right)\right)$ and $\Gamma_{T}\left[x\left(t\right),a\right]\;\circ \;\Gamma_{T}\left[x\left(t\right),-a\right]=\Gamma_{T}\left[\Gamma_{T}\left[x\left(t\right),a\right]-a\right]=\Gamma_{T}\left[x\left(t\right),a-a\right]=\Gamma_{T}\left[x\left(t\right),0\right]=\Gamma_{\epsilon }\left[x\left(t\right)\right]=x\left(t\right)$. The other compositions are reported in the Cayley table (see Table 1); for more details, see Appendix A.4.
2. The vertical reflection operation is defined as follows: $x^{\prime }\left(t\right)=\Gamma_{R}\left[x\left(t\right),\tau \right]=x\left(-t+2\tau \right)$, where τ is a delay. The signal x(t) is invariant under vertical reflection if it satisfies the following: $x^{\prime }\left(t\right)=x\left(t\right)$. Note that $\Gamma_{R}\left[x\left(t\right),\tau \right]\;\circ \;\Gamma_{R}\left[x\left(t\right),-\tau \right]=\Gamma_{R}\left[x\left(t\right),0\right]=\Gamma_{\epsilon }\left[x\left(t\right)\right]=x\left(t\right)$ and $\Gamma_{R}\left[x\left(t\right),f\right]\;\circ \;\Gamma_{R}\left[x\left(t\right),e\right]=x\left(-t+2f-2e\right)$. The other compositions are indicated in the Cayley table (see Table 1); for more details, see Appendix A.4.
3. The inversion operation is defined as follows: $x^{\prime }\left(t\right)=\Gamma_{I}\left[x\left(t\right),\tau \right]=-x\left(-t+2\tau \right)$, where τ is a delay. The signal x(t) is invariant under inversion if it satisfies the following: $x^{\prime }\left(t\right)=x\left(t\right)$. Note that $\Gamma_{I}\left[x\left(t\right),\tau \right]\;\circ \;\Gamma_{I}\left[x\left(t\right),-\tau \right]=\Gamma_{I}\left[x\left(t\right),0\right]=\Gamma_{\epsilon }\left[x\left(t\right)\right]=x\left(t\right)$ and $\Gamma_{I}\left[x\left(t\right),h\right]\;\circ \;\Gamma_{I}\left[x\left(t\right),g\right]=x\left(t+2h-2e\right)$. The other compositions are indicated in the Cayley table (see Table 1); for more details, see Appendix A.4.
4. The glide reflection operation is defined as follows: $x^{\prime }\left(t\right)=\Gamma_{G}\left[x\left(t\right),\tau \right]=-x\left(t-\tau \right)$, where τ is a delay. The signal x(t) is invariant under glide reflection if it satisfies the following: $x^{\prime }\left(t\right)=x\left(t\right)$. Note that $\Gamma_{G}\left[x\left(t\right),\tau \right]\;\circ \;\Gamma_{G}\left[x\left(t\right),-\tau \right]=\Gamma_{G}\left[x\left(t\right),0\right]=\Gamma_{\epsilon }\left[x\left(t\right)\right]=x\left(t\right)$ and $\Gamma_{G}\left[x\left(t\right),d\right]\;\circ \;\Gamma_{G}\left[x\left(t\right),c\right]=x\left(t-\left(d+c\right)\right)$. The other compositions are indicated in the Cayley table (see Table 1); for more details, see Appendix A.4.

The set of compositions of signal isometries, which can be assembled in the Cayley table (see Table 1), forms the group of isometries (or symmetry group).

### 2.2. Symmetry Group

The symmetry group of a signal is the group of all isometries under which the signal is invariant. A signal with no symmetry will be invariant only in the identity operation. The group is not merely a set of operations; it possesses an algebraic structure and is equipped with an internal composition law. The group is defined by the following axioms:

(i) If the set contains the elements $\Gamma_{i}$ and $\Gamma_{j}$, then it contains the products $\Gamma_{i}\;\circ \;\Gamma_{j}$ and $\Gamma_{i}\;\circ \;\Gamma_{i}=\Gamma_{i}^{2}$;
(ii) The composition law is associative: $\left(\Gamma_{i}\;\circ \;\Gamma_{j}\right)\;\circ \;\Gamma_{k}=\Gamma_{i}\;\circ \;\left(\Gamma_{j}\;\circ \;\Gamma_{k}\right)$;
(iii) The set contains the identity or neutral element $\Gamma_{\epsilon }$ such that $\Gamma_{i}\;\circ \;\Gamma_{\epsilon }=\Gamma_{\epsilon }\;\circ \;\Gamma_{i}=\Gamma_{i}$;
(iv) If the set contains the element $\Gamma_{i}$, then it contains the inverse element $\Gamma_{i}^{-1}$ that satisfies $\Gamma_{i}\;\circ \;\Gamma_{i}^{-1}=\Gamma_{i}^{-1}\;\circ \;\Gamma_{i}=\Gamma_{\epsilon }$.

**Figure 2:**
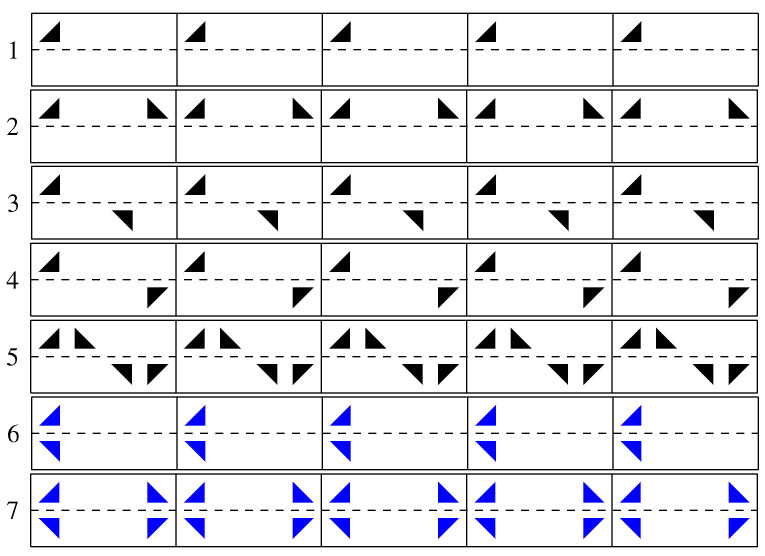
Seven types of friezes with the pattern 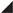. Friezes 1, 2, 3, 4, and 5 possess a periodic signal equivalent with the same symmetries. Friezes 6 and 7 (with the blue patterns) do not have equivalent periodic signals because the generated functions are surjective, whereas signals are bijective.

There are several families of groups; here, only the cyclic group, the dihedral group, and the frieze group are introduced:

- The simplest family of groups is the family of cyclic groups. This is the group that uses a single generating operation Γ (also called the generator). A cyclic group of order *n* is a group of the form $C_{n}=<\Gamma \;|\;\Gamma^{n}=\Gamma_{\epsilon }>$ with a specific operation $\Gamma^{n}=\Gamma_{\epsilon }$, where Γ is repeated *n* times (<|> is not an inner product. This is a representation from group theory where the term at the left of the bar corresponds to the generator and where the term at the right of the bar corresponds to operations see [21,22].). The infinite cyclic group is obtained with a single generating operation with no particular relations, $C_{\infty }=<\Gamma |>$, and the elements of the group are $\left\{\ldots \Gamma^{-3},\Gamma^{-2},\Gamma^{-1},\Gamma^{0},\Gamma^{1},\Gamma^{2},\Gamma^{3},\ldots \right\}$;
- The second simplest family of groups is the family of dihedral groups. This is the group that uses two generating operations: $\Gamma_{i}$ and $\Gamma_{j}$. The dihedral group of order 2n is of the form $D_{2n}=\langle \Gamma_{i},\Gamma_{j}\;|\;\Gamma_{i}^{n}=\Gamma_{j}^{2}=\Gamma_{\epsilon },\Gamma_{j}\Gamma_{i}=\Gamma_{i}^{-1}\Gamma_{j}\rangle$ with two specific operations $\Gamma_{i}^{n}=\Gamma_{j}^{2}=\epsilon$ and $\Gamma_{j}\;\circ \;\Gamma_{i}=\Gamma_{i}^{-1}\;\circ \;\Gamma_{j}$. The infinite dihedral group is $D_{\infty }=\langle \Gamma_{i},\Gamma_{j}\;|\;\Gamma_{j}^{2}=\Gamma_{\epsilon },\Gamma_{j}\circ \Gamma_{i}=\Gamma_{i}^{-1}\;\circ \;\Gamma_{j}\rangle$;
- The third group presented is the symmetry group of friezes (A frieze is an infinitely long strip of finite height on which periodic patterns are printed). Examples of friezes are shown in Figure 2. This group includes cyclic and dihedral groups, which are said to be subgroups of the frieze group. As we will see later, the symmetry group of friezes encompasses the symmetry group of periodic signals, which is a subgroup of the frieze symmetry group. As indicated in Table A2 in Appendix A.3, the symmetry group of friezes is based on five isometries (translation, vertical reflection, glide reflection, inversion, and horizontal reflection). The group of periodic signals, on the other hand, is based on only four isometries (translation, vertical reflection, glide reflection, and inversion), with horizontal reflection $\Gamma_{H}\left[x\left(t\right)\right]$ being forbidden. Indeed, the use of this last isometry leads to a frieze with two values for the same position on the horizontal axis (see friezes 6 and 7 with blue patterns in Figure 2), which is equivalent to a “surjection” for signals, whereas signals are by definition bijective functions. A direct consequence of the prohibition on using horizontal reflection alone is that the total number of types of periodic signals is five, compared to seven for friezes. It is easy to see that the sixth and seventh friezes (see the blue patterns in Figure 2) cannot have equivalent periodic signals.

Here are a few examples of subgroups of the symmetry group of periodic signals G:

(a) The translation group $G_{T}$ uses a single generator: the translation $\Gamma_{T}$. It is a subgroup of the symmetry group of periodic signals G: $G_{T}=C_{\infty }=<\Gamma_{T}\;|\;>$ where $\Gamma_{T}\left[x\left(t\right),\tau \right]=x\left(t-\tau \right)$ is the translation to the right by the delay τ;
(b) The reflection group $G_{R}$ uses two generators: the translation $\Gamma_{T}$ and the vertical reflection $\Gamma_{R}$. It is a subgroup of the symmetry group of periodic signals G: $G_{R}=D_{\infty }=\langle \Gamma_{T},\Gamma_{R}\;|\;\Gamma_{R}^{2}=\Gamma_{\epsilon },\Gamma_{R}\circ \Gamma_{T}=\Gamma_{T}^{-1}\circ \Gamma_{R}\rangle$ where $\Gamma_{\epsilon }$ is the identity operation, $\Gamma_{R}\left[x\left(t\right),\tau \right]=x\left(-t+2\tau \right)$ is the vertical reflection;
(c) The inversion group $G_{I}$ uses two generators: the translation $\Gamma_{T}$ and the inversion $\Gamma_{I}$. It is a subgroup of the symmetry group of periodic signals G: $G_{I}=D_{\infty }=\langle \Gamma_{T},\Gamma_{I}\;|\;\Gamma_{I}^{2}=\Gamma_{\epsilon },\Gamma_{I}\circ \Gamma_{T}=\Gamma_{T}^{-1}\circ \Gamma_{I}>$ where $\Gamma_{I}\left[x\left(t\right),\tau \right]=-x\left(-t+2\tau \right)$ is the inversion;
(d) The glide reflection group $G_{G}$ uses a single generator: the glide reflection $\Gamma_{G}$. It is a subgroup of the symmetry group of periodic signals G: $G_{G}=C_{\infty }=<\Gamma_{G}\;|\;>$ where $\Gamma_{G}\left[x\left(t\right),\tau \right]=-x\left(t-\tau \right)$ is the glide reflection to the right by the delay τ;
(e) The glide reflection and inversion group $G_{GI}$ (or the glide reflection and vertical reflection group $G_{GR}$) uses two generators: the glide reflection $\Gamma_{G}$ and the inversion $\Gamma_{I}$. It is a subgroup of the symmetry group of periodic signals G: $G_{GI}=D_{\infty }=\langle \Gamma_{G},\Gamma_{I}\;|\;\Gamma_{I}^{2}=\epsilon ,\Gamma_{I}\circ \Gamma_{G}=\Gamma_{G}^{-1}\circ \Gamma_{I}\rangle$.

### 2.3. Generation of Signals

The generation of friezes or signals can be achieved using an iterative process (see Equation (1)). By utilizing compositions of isometries of type j∈{T,R,I,G} and a generator pattern (Do not confuse the group generator, which is an isometry $\Gamma_{j}$, with the generator pattern $x_{0}\left(t\right)$, which is the signal serving as the basic building block to construct a more complex signal) $x_{0}\left(t\right)$, it is possible to construct symmetric signals with as many steps *n* as desired, using the following generating equation:

(1) $$x_{n}\left(t\right)=x_{n-1}\left(t\right)+\Gamma_{j}\left[x_{n-1}\left(t\right),\tau \right],$$

where τ is a delay.

The choices of the generator pattern $x_{0}\left(t\right)$, the number of iterations *n* and the considered isometries $\Gamma_{j}$, will determine the properties of the generated signal. We can already assert that if the number of iterations *n* is infinite, then the signal will be periodic regardless of the type of isometry considered among the four previously mentioned. However, not all signals generated by Equation (1) will necessarily be symmetric; a judicious choice of the generator pattern, the number of iterations, and the considered isometries will be required to achieve symmetry.

To complete the description of the generator pattern, consider the left endpoint $x_{0}\left(t_{l}\right)$ and the right endpoint $x_{0}\left(t_{r}\right)$ of the generator pattern $x_{0}\left(t\right)$. In the most general case, the two endpoints are different, i.e., $x_{0}\left(t_{l}\right)\neq x_{0}\left(t_{r}\right)$. For these conditions, if the generator pattern $x_{0}\left(t\right)$ is continuous, then the signal $x_{1}\left(t\right)$ at step n=1 is continuous, i.e., $x_{0}\left(t_{r}^{-}\right)=x_{0}^{\prime }\left(t_{r}^{+}\right)$ if and only if $x_{0}^{\prime }=\Gamma_{R}\left[x_{0}\left(t\right),\tau \right]$, where τ is chosen to ensure equality. Additionally, if the derivatives are equal, i.e., ${\dot{x}}_{0}\left(t_{r}^{-}\right)={\dot{x}}_{0}^{\prime }\left(t_{r}^{+}\right)$ then the inversion operation $x_{0}^{\prime }=\Gamma_{I}\left[x_{0}\left(t\right),\tau \right]$ can also be used. If $x_{0}\left(t_{l}\right)=x_{0}\left(t_{r}\right)$, continuity is ensured for all four isometries. Finally, if the generator pattern already possesses symmetry properties, the corresponding Cayley table simplifies as indicated in the Appendix A.3 in Table A3 when the generator pattern ∩ is even and in Table A4 when the generator pattern ∩∪ is odd.

**Figure 3:**
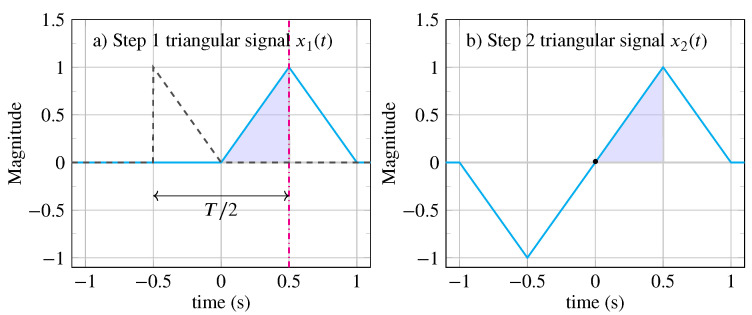
A triangular signal of duration T=2 s with a generator pattern $x_{0}\left(t\right)=2t\times Rect_{T/4}\left(t-T/8\right)$ of duration T/4 whose area under the curve is colored in blue. (**a**) Step 1: The signal $x_{1}\left(t\right)$ is obtained using a vertical reflection with the reflection axis located at t=0.5 s (vertical magenta dash-dotted line). $x_{0}\left(-t\right)$ is reported with black dashed line. A delay of T/2 separates $x_{1}\left(t\right)$ and $x_{0}\left(-t\right)$. (**b**) Step 2: The signal $x_{2}\left(t\right)$ is obtained using an inversion with the inversion center located at t=0 s.

**Example** **1.** *Consider the generator signal $x_{0}\left(t\right)=2t\times Rect_{T/4}\left(t-T/8\right)$ with a duration of T/4. Several symmetric signals (illustrated in Figure 3) can be generated depending on the number of steps chosen:*

*1.*  *The signal at step n=1 is given by the following: $x_{1}\left(t\right)=x_{0}\left(t\right)+\Gamma_{R}\left[x_{0}\left(t\right),T/4\right]$ where $\Gamma_{R}\left[x_{0}\left(t\right),T/4\right]$$=x_{0}\left(-t+T/2\right)$. Substituting the expression for $\Gamma_{R}\left[x_{0}\left(t\right),T/4\right]=x_{0}\left(-t+T/2\right)$, we obtain $x_{1}\left(t\right)=2t\times Rect_{T/4}\left(t-T/8\right)+2\left(-t+T/2\right)\times Rect_{T/4}\left(-t+3T/8\right)$;*
*2.*  *The signal at step n=2 is given by the following: $x_{2}\left(t\right)=x_{1}\left(t\right)+\Gamma_{I}\left[x_{1}\left(t\right),0\right]=x_{1}\left(t\right)-x_{1}\left(-t\right)$. Substituting the expressions for $x_{1}\left(t\right)$, we obtain $x_{2}\left(t\right)=x_{0}\left(t\right)+x_{0}\left(-t+T/2\right)-x_{0}\left(-t\right)-x_{0}\left(t-T/2\right)$. Finally, expressing $x_{2}\left(t\right)$ in terms of the generator pattern $x_{0}\left(t\right)$, we obtain $x_{2}\left(t\right)=2t\times Rect_{T/4}\left(t-T/8\right)+2\left(-t+T/2\right)\times Rect_{T/4}\left(-t+3T/8\right)+2t\times Rect_{T/4}\left(-t-T/8\right)+2\left(-t-T/2\right)\times Rect_{T/4}\left(t-3T/8\right)$.*

### 2.4. Classification

Taking into account all the previously presented information, it is possible to propose alternative classifications P and Q to the F-classification. These classifications for signals with global symmetry are reported in Table 2. The P-classification is dedicated to periodic signals, while the Q-classification is dedicated to non-periodic signals. To simplify these two classifications, we propose a binary coding scheme for the different classes as indicated in Table 3.

For periodic signals of P-classification, we have seen that there are five types, and they correspond to a subgroup of the symmetry group of friezes. For class $P_{1}$, signals are invariant under a single isometry: translation ($\Gamma_{T}$). For classes $P_{3}$, $P_{5}$, and $P_{9}$, signals are invariant under two isometries (see Table 2). For class $P_{15}$, signals are invariant under all four isometries.

For non-periodic symmetric signals of Q-classification, the number of classes is limited to two: $Q_{2}$ and $Q_{4}$. Only improper isometries (vertical reflection $\Gamma_{R}$ and inversion $\Gamma_{I}$) are used to verify invariance. These signals are associated with the dihedral group $D_{2}$, as invariance by translation and glide reflection are no longer possible. While non-periodic symmetric signals belong to specific classes ($Q_{2}$ or $Q_{4}$), they may possess local symmetries. This point will be detailed further.

**Figure 4:**
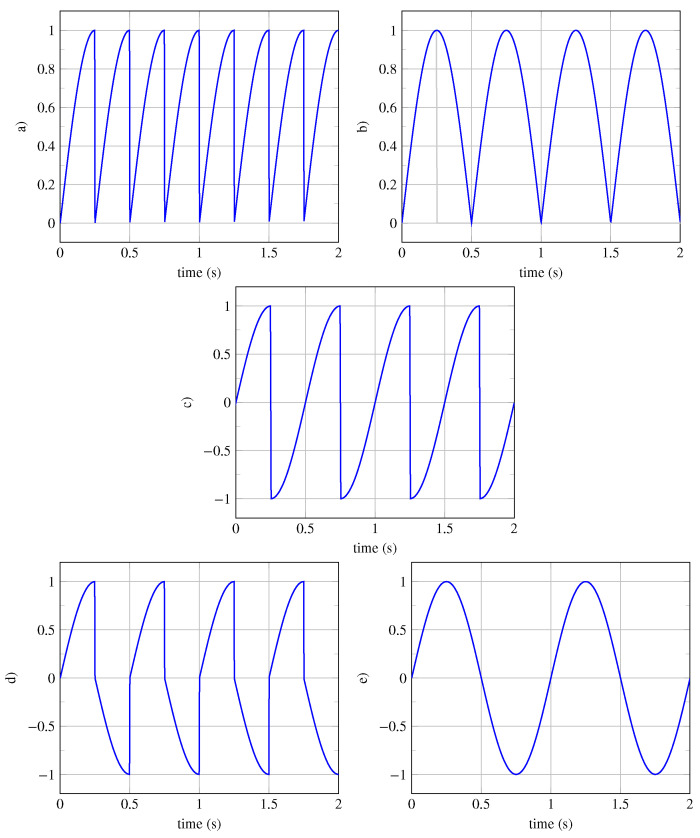
P-classification of periodic signals. (**a**) Signal of class $P_{1}$ invariant under translation only. (**b**) Signal of class $P_{3}$ invariant under translation and vertical reflection. (**c**) Signal of class $P_{5}$ invariant under translation and inversion. (**d**) Signal of class $P_{9}$ invariant under translation and glide reflection. (**e**) Signal of class $P_{15}$ invariant under all four isometries.

**Example** **2.** *Let us consider the generator pattern defined by the following: $x_{0}\left(t\right)=Rect_{T/4}\left(t-T/8\right)\times \sin\left(\frac{2\pi }{T}t\right)$. We aim to generate a periodic signal of class $P_{1}$, which is invariant under translation only. The resulting periodic signal x(t) is represented in Figure 4a. The periodic signal x(t) is calculated using the generator equation (see Equation (1)) when n→∞:*

$$x\left(t\right)=\lim_{n\to \infty }x_{n}\left(t\right)$$

*with*

$$x_{n}\left(t\right)=x_{n-1}\left(t\right)+\Gamma_{T}\left[x{\left(t\right)}_{n-1},{\left(-1\right)}^{n+1}\times 2^{n-1}T/4\right]$$

$$\;\;=x_{n-1}\left(t\right)+x_{n-1}\left(t-\left({\left(-1\right)}^{n+1}\times 2^{n-1}T/4\right)\right).$$

*The previous generating equation is reported thereafter for the first three iterations:*

$$x_{1}\left(t\right)=x_{0}\left(t\right)+x_{0}\left(t-\left({\left(-1\right)}^{2}\times 2^{0}T/4\right)\right)=x_{0}\left(t\right)+x_{0}\left(t-T/4\right),$$

$$x_{2}\left(t\right)=x_{1}\left(t\right)+x_{1}\left(t-\left({\left(-1\right)}^{3}\times 2^{1}T/4\right)\right)=x_{0}\left(t\right)+x_{0}\left(t+2T/4\right),$$

$$x_{3}\left(t\right)=x_{2}\left(t\right)+x_{2}\left(t-\left({\left(-1\right)}^{4}\times 2^{2}T/4\right)\right)=x_{0}\left(t\right)+x_{0}\left(t-4T/4\right).$$

*Once the signal $x\left(t\right)=\lim_{n\to \infty }x_{n}\left(t\right)$ of type $P_{1}$ is obtained, we easily verify its invariance under translation $\Gamma_{T}\left[x\left(t\right),kT\right]=x\left(t\right)$, k∈N. The other four types of periodic signals are shown in Figure 4b–e.*

**Example** **3.** *Let us take the case where the generating pattern is defined by $x_{0}\left(t\right)=2t\times Rect_{T/4}\left(t-T/8\right)$ (pattern colored in blue in Figure 3a), with a duration of T/4=1/2 s, and create a non-periodic signal of type $Q_{2}$ that is invariant under mirror reflection with a duration of 1 s. This signal $x\left(t\right)=x_{1}\left(t\right)$, shown in Figure 3a, is calculated from the generating equation $x\left(t\right)=x_{n}\left(t\right)$ for n=1:*

$$x_{1}\left(t\right)=x_{0}\left(t\right)+\Gamma_{R}\left[x_{0}\left(t\right),T/4\right]=x_{0}\left(t\right)+x_{0}\left(-t-T/2\right)).$$

*Its invariance under mirror reflection $\Gamma_{T}\left[x\left(t\right),T/4\right]=x\left(t\right)$ is easily verified at the position of the reflection axis at t=T/4=1/2 s.*

**Remark** **1.**

- *When the invariance is no longer global, there is an infinite number of non-periodic symmetric signals;*
- *The use of the generating Equation (1) does not guarantee that a non-periodic signal will be symmetric. Indeed, for a non-periodic signal to be symmetric, the last isometry used in the iterative process (if there are several) must be an improper isometry (vertical reflection or inversion) and the moment when the isometry operates must correspond to a symmetry element (axis of reflection or center of inversion).*

## 3. Symmetry Indicators

Now that we know how to generate symmetric signals and have established a classification, we must address the question of analyzing symmetric signals, particularly how to measure the level of symmetry present in a signal. To answer this question, we will begin with the work of [10], which discusses the concepts of “symmetropy” (Symmetry (Symmetria) and change (tropia)) and “symmentropy” (Symmetry (Symmetria) and entropy/transformation (entropia)) in palindromic signals. In [10], the authors demonstrated the potential of these descriptors to evaluate different levels of symmetry in discrete sequences such as DNA or binarized fractional Brownian motion. Since the sequences were discrete in [10], the metrics were calculated point by point. Because we are working with continuous signals, the mathematical framework here will be continuous.

### 3.1. Symmetrometry

We have previously seen that there are only four possible isometries for generating and classifying signals. We have also seen that a judicious choice of the generating pattern $x_{0}\left(t\right)$, the number *n* of iterations, and the isometries $\Gamma_{j}\left[x\left(t\right),\tau \right]$ can lead to the generation of symmetric signals. A way to measure the level of symmetry present in a signal could consist of measuring the similarity between the studied signal x(t) and its different transformed versions $x^{\prime }\left(t\right)=\Gamma_{j}\left[x\left(t\right),\tau \right]$ with j∈T,R,I,G for each of the four possible isometries. The sum of these four measurements would then result in the **global symmetrometry** (Symmetry (Symmetria) and measurement (metron)) S, a concept very close to the symmetropy proposed in [10].

Let $d_{j}\left(\tau \right)$, the average deviation between the signal x(t) under study and its transformed version $x^{\prime }\left(t\right)=\Gamma_{j}\left[x\left(t\right),\tau \right]$ for different values of the delay τ, be defined for finite average power signals as follows:

(2) $$d_{j}\left(\tau \right)=\lim_{T\to \infty }\frac{1}{T}\int_{-T/2}^{T/2}\left|x\left(t\right)-\Gamma_{j}\left[x\left(t\right),\tau \right]\right|dt,$$

and for finite energy signals by

(3) $$d_{j}\left(\tau \right)=\frac{1}{T}\int_{0}^{T}\left|x\left(t\right)-\Gamma_{j}\left[x\left(t\right),\tau \right]\right|dt$$

with j∈T,R,I,G. Let us recall the following relations: $\Gamma_{T}\left[x\left(t\right),\tau \right]=x\left(t-\tau \right)$, $\Gamma_{R}\left[x\left(t\right),\tau \right]=x\left(-t+2\tau \right)$, $\Gamma_{I}\left[x\left(t\right),\tau \right]=-x\left(-t+2\tau \right)$, and $\Gamma_{G}\left[x\left(t\right),\tau \right]=-x\left(t-\tau \right)$, where τ is a delay. When the function $d_{j}\left(\tau \right)$ is zero for a fixed value of τ, the signal x(t) and its isometric version $x^{\prime }\left(t\right)$ are identical. If the position τ corresponds to a symmetry element, such as a reflection axis, then the signals x(t) and $x^{\prime }\left(t\right)$ are symmetric. This is the case, for example, for the signal x(t)=exp(−|t−γ|)+exp(−|t+γ|) when t=±γ, which corresponds to two reflection axes.

The calculation of the integral of this distance $d_{j}\left(\tau \right)$ allows us to obtain a scalar that indicates the level of similarity between the two signals x(t) and $x^{\prime }\left(t\right)$. It seems more appropriate to construct a metric that equals one when the similarity is maximal. For each type of isometry, the **average typed symmetrometry** $S_{j}$ is calculated, depending on the nature of the signal, as follows:

(4) $$S_{j}=\lim_{T\to \infty }\frac{1}{T}\int_{-T/2}^{T/2}\sigma_{j}\left(\tau \right)d\tau ,$$

or

(5) $$S_{j}=\frac{1}{T}\int_{0}^{T}\sigma_{j}\left(\tau \right)d\tau ,$$

with the **typed symmetrometries** $\sigma_{j}\left(\tau \right)$ defined as

(6) $$\sigma_{j}\left(\tau \right)=1-d_{j}^{*}\left(\tau \right),$$

where $d_{j}^{*}\left(\tau \right)=d_{j}\left(\tau \right)/\max\left(d_{j}\left(\tau \right)\right)$ is the normalized distance.

To account for the fact that there are two different categories of signals, the category of periodic signals whose invariance depends on four isometries and the category of non-periodic signals whose invariance depends on only two isometries, the coefficients $\beta_{j}$ are used to weight the average typed symmetrometries. The **global symmetrometry** is then defined as follows:

(7) $$S=\frac{1}{2}\sum_{j}\left(S_{j}\times \beta_{j}\right),$$

where $\beta_{j}=\sigma_{j}^{*}=max\left(\sigma_{j}\right)$ with j∈T,R,I,G. Note that the coefficient 1/2 ensures that the most symmetric signal, presenting invariances for all four types of isometries, reaches a maximum **global symmetrometry**, S=1. In this case, the four average typed symmetrometries are equal: $S_{T}=S_{R}=S_{I}=S_{G}=1/2$.

For non-periodic symmetric signals, only the invariances associated with improper isometries need to be considered, and in these conditions, we impose $\beta_{T}=\beta_{G}=0$. For non-periodic symmetric signals, the global symmetrometry cannot exceed S≤1/2.

To complement the global symmetrometry indicator, based on the average of the typed symmetrometries $\sigma_{j}$, we propose another indicator based on the average of the maxima of the typed symmetrometries $\sigma_{j}^{*}=max\left(\sigma_{j}\right)$. The indicator φ is defined as the average of the **maxima of the typed symmetrometries** $\sigma_{j}^{*}$ and is written as follows:

(8) $$\varphi =\frac{1}{4}\sum_{j}\sigma_{j}^{*}.$$

**Figure 5:**
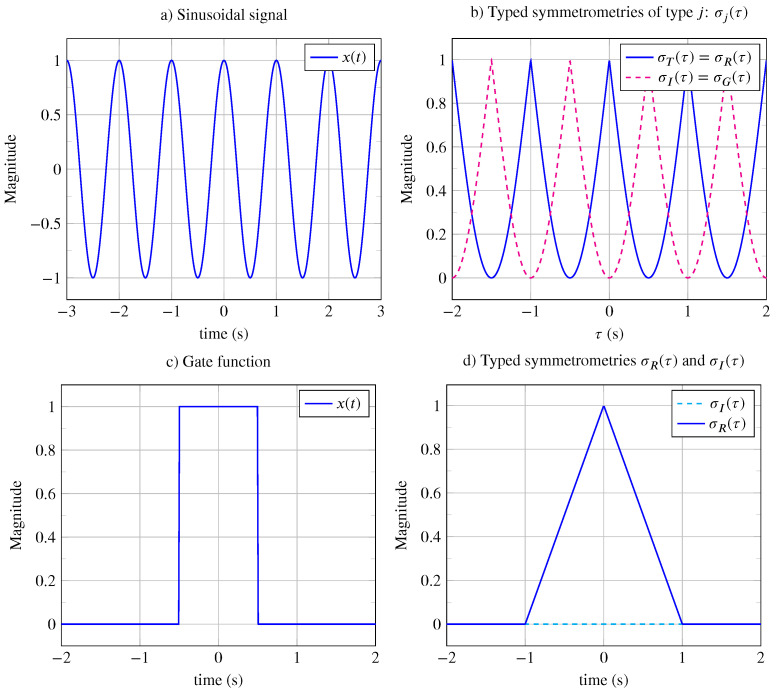
Symmetry indicators: (**a**) Sinusoidal signal. (**b**) Typed symmetrometries $\sigma_{T}\left(\tau \right)=\sigma_{R}\left(\tau \right)$ and $\sigma_{I}\left(\tau \right)=\sigma_{G}\left(\tau \right)$ (dashed line) obtained from sinusoidal signal. The maximum of typed symmetrometries are equal to one. (**c**) Gate function. (**d**) Typed symmetrometry $\sigma_{R}\left(\tau \right)$ obtained from the gate function is a triangular function, $\sigma_{I}\left(\tau \right)=0$ (dashed line).

**Example** **4.** *Let us take the example of the cosinusoidal signal with x(t)=cos(2πt/T) of period T=1 s, which is shown in Figure 5a, over a duration of 6 s. Since the signal x(t) is periodic, the calculation of the distance $d_{T}\left(\tau \right)$ between the signal and its translated version is computed over the period T:*

$$d_{T}\left(\tau \right)=\frac{1}{T}\int_{0}^{T}|x\left(t\right)-\Gamma_{T}\left[x\left(t\right),\tau \right]|dt=\frac{1}{T}\int_{0}^{T}|x\left(t\right)-x\left(t-\tau \right)|dt=\frac{1}{T}\int_{0}^{T}\left|cos\left(2\pi t/T\right)-cos\left(2\pi \left(t-\tau \right)/T\right)\right|dt.$$

*After calculation, we obtain the following:*

$$d_{T}\left(\tau \right)=\frac{4}{\pi }\left|\sin\left(\frac{2\pi \tau }{2T}\right)\right|,$$

$$d_{T}^{*}\left(\tau \right)=d_{T}\left(\tau \right)/max\left(d_{T}\left(\tau \right)\right)=\left|\sin\left(\frac{\pi \tau }{T}\right)\right|.$$

*The typed translation symmetrometry shown in Figure 5b is also a periodic function, which is maximal when the signal x(t) and its transformed version $x^{\prime }\left(t\right)=\Gamma_{T}\left[x\left(t\right),\tau \right]$ are superimposed (i.e., for all periods T=1 s) and is given by the following:*

$$\sigma_{T}\left(\tau \right)=1-d_{T}^{*}\left(\tau \right),$$

and as shown in Figure 5b, $\sigma_{T}^{*}=1$.

*Since the typed translation symmetrometry is periodic, it is calculated over its period T and is given by the following:*

$$S_{T}=\frac{1}{T}\int_{0}^{T}\left(1-d_{T}^{*}\left(\tau \right)\right)d\tau =1-\frac{1}{T}\int_{0}^{T}d_{T}^{*}\left(\tau \right),$$

$$S_{T}=1-\frac{1}{T}\int_{0}^{T}\left|\sin\left(\frac{\pi \tau }{T}\right)\right|d\tau =1-\frac{4T}{2\pi T}=1-\frac{2}{\pi }\approx 0.36$$

It can be shown that $d_{R}\left(\tau \right)=d_{T}\left(\tau \right)$, $d_{I}\left(\tau \right)=d_{G}\left(\tau \right)=d_{T}\left(\tau -T_{0}/2\right)$, and φ=1 with $\sigma_{T}^{*}=\sigma_{R}^{*}=\sigma_{I}^{*}=\sigma_{G}^{*}=1$.

*Finally, for the cosinusoidal signal, the global symmetrometry is the following:*

$$S=\frac{1}{2}\left(4-\frac{8}{\pi }\right)\approx 0.72$$

**Example** **5.** *Let us consider the finite energy signal defined by $x\left(t\right)=Rect_{T}\left(t\right)$ with T=1 s and for t∈−L/2,L/2 with L=4 s. It is a non-periodic even signal x(t)=x(−t) with global symmetry, and it has a reflection axis corresponding to the vertical axis. Let us calculate the typed vertical reflection symmetrometry by first calculating $d_{R}\left(\tau \right)$:*

$$d_{R}\left(\tau \right)=\frac{1}{L}\int_{-L/2}^{L/2}|x\left(t\right)-\Gamma_{R}\left[x\left(t\right),\tau \right]|dt=\frac{1}{L}\int_{-L/2}^{L/2}|x\left(t\right)-x\left(-t+\tau \right)|dt=\frac{1}{L}\int_{-L/2}^{L/2}\left|Rect_{T}\left(t\right)-Rect_{T}\left(-t+\tau \right)\right|dt,$$

*where L is the support of $|Rect_{T}\left(t\right)-Rect_{T}\left(-t+\tau \right)|$.*

*After the calculation, we find the following:*

$$d_{R}\left(\tau \right)=2\frac{\left|t\right|}{T}+\left(1+sign(|t|-T)\right)$$

$$\sigma_{R}\left(\tau \right)=1-d_{R}^{*}\left(\tau \right)=tria_{T}\left(t\right),$$

with $\sigma_{R}^{*}=1$.

*The average vertical reflection symmetrometry is the following:*

$$S_{R}=\frac{1}{L}\int_{-L/2}^{L/2}\sigma_{R}\left(\tau \right)d\tau =1/4.$$

The average typed symmetrometries are the following: $S_{R}=1/4$ and $S_{I}=0$, and φ=1/4 with $\sigma_{R}^{*}=1$ and $\sigma_{I}^{*}=0$.

*The global symmetrometry is the following:*

S=1/8,

*with $\beta_{T}=\beta_{G}=0$.*

**Remark** **2.**

- *The average distance $d_{j}\left(\tau \right)$ given in Equation (2) is based on the so-called $L_{1}$ norm; however, other norms like the $L_{2}$ norm (or others) could also be used;*
- *The previously introduced metrics quantify isometric similarities by summing, for example, the four average typed symmetrometries. However, these metrics do not account for the relative proportions between each average typed symmetrometry. A way to account for the distribution of average typed symmetrometries is to calculate the “symmentropy”.*

### 3.2. Symmentropy

A measure of the informational diversity of the different types of symmetry can be obtained by transforming the average typed symmetrometries $S_{j}$ into probability measures $p_{j}=\beta_{j}\times S_{j}/S$ and then calculating the symmentropy (Symmetry (Symmetria) and entropy/transformation (entropia)) using the same definition introduced in [10] as follows:

(9) $$E=-\sum_{j}p_{j}\times \log_{4}\left(p_{j}\right),$$

where j∈T,R,I,G and $\beta_{j}=1:\forall :j$. In order to obtain maximum symmetry equal to unity, a logarithm in base 4 is proposed. Maximum symmetry is obtained when equiprobability exists. Note that for non-periodic signals, $\beta_{T}=\beta_{G}=0$, and to allow comparison with periodic signals, the 4-based logarithmic is maintained.

**Remark** **3.** *It can be shown that for the cosinusoidal signal, the typed symmetrometries are equal, and there is an equal distribution $p=p_{j}=\frac{{\bar{\sigma }}_{j}}{\bar{\sigma }}=\frac{1}{4}$. The symmentropy is maximal and equals E=1. For the rectangular signal, the symmentropy equals E=0 because $S_{R}=1$ and $S_{I}=0$.*

### 3.3. Distortion Indicators: Distorsymmetry

Previously, in many applications, it has been emphasized that one way to characterize a system is by measuring the distortion of signals at the output of the system. Here, we propose one indicator that measures the distortion experienced by a signal, which is not based on power measurement but on symmetry.

This indicator of **distorsymmetry** (Distortion and symmetry (symmetria)) θ is defined as follows:

(10) θ=2(1−φ).

This indicator is based on the symmetrometry φ, itself based on the maxima of the typed symmetrometries $\sigma_{j}^{*}$. When the maximal typed symmetrometries are maximal, the level of global symmetry is maximal and the distortion is minimal, θ=0.

### 3.4. Classification Indicators: Isorithm and Symtaxis

To facilitate the classification of symmetric signals, we propose two indicators calculated from the integer values of the maxima of the typed symmetrometries $\left\lfloor \sigma_{i}^{*}\right\rfloor$. The first indicator measures the number of different types of invariance, and the second calculates the class membership. The indicator **isorithm** (isorithm: measurement (metron), equal (iso), and number (artimos)) ϕ is defined as follows:

(11) $$\phi =\sum_{i=1}^{4}\left\lfloor \sigma_{i}^{*}\right\rfloor .$$

This indicator counts the number of different invariances and compares the classes between them. As we will see later with examples, the isorithm ϕ for class $P_{15}$ signals is four times higher than for class $P_{1}$ signals and two times higher than for class $P_{3}$ signals.

The second indicator is symtaxis (symtaxis: Ranking (taxis), symmetry (symmetria)), and it is defined as follows:

(12) $$C=\sum_{i=1}^{4}2^{i-1}\left\lfloor \sigma_{i}^{*}\right\rfloor$$

with $\sigma_{1}^{*}=\sigma_{T}^{*}$, $\sigma_{2}^{*}=\sigma_{R}^{*}$, $\sigma_{3}^{*}=\sigma_{I}^{*}$, $\sigma_{4}^{*}=\sigma_{G}^{*}$. For class $P_{1}$ signals, we have $\lfloor \sigma_{1}^{*}\rfloor =1$ and $\left\lfloor \sigma_{2}^{*}\right\rfloor =\left\lfloor \sigma_{3}^{*}\right\rfloor =\left\lfloor \sigma_{4}^{*}\right\rfloor =0$, and C=1. For other classes, we obtain 3,5,9,15 for $P_{3},P_{5},P_{9},P_{15}$, respectively.

### 3.5. Remarks

- All indicators can be calculated directly from the numerical data of the signals, replacing integrals with sums, so knowing the analytical expression of the signals is no longer necessary.
- To measure the degree of similarity between the original signal and its isometric version, several metrics can be used. Here, **another type of metric** is defined as follows:
  $$\sigma_{j}=\lim_{T\to \infty }\frac{1}{T}\int_{-T/2}^{T/2}\gamma_{j}\left(\tau \right)dt,$$
  $$\gamma_{j}\left(\tau \right)=\rho \left(\tau \right)+\left|\rho \left(\tau \right)\right|,$$
  $$\rho_{j}\left(\tau \right)=\lim_{T\to \infty }\frac{1}{T}\int_{-T/2}^{T/2}x\left(t\right)\times \Gamma_{j}\left[x\left(t\right),\tau \right]dt,$$
  being a correlation function. Since this metric is based on correlation measurement, it is likely to be less sensitive to noise than the metric $d_{j}\left(\tau \right)$.
- The various proposed symmetry indicators are based on a similarity measure. This similarity measure is based on the difference between the signal x(t) and its isometric version $x^{\prime }\left(t\right)$. For example, in the case of translation and in the presence of noise n(t), the noisy signal is written y(t)=x(t)+n(t) and its noisy isometric version is written $y^{\prime }\left(t\right)=x\left(t-\tau \right)+n\left(t-\tau \right)$. The difference then becomes $y\left(t\right)-y^{\prime }\left(t\right)=x\left(t\right)-x\left(t-\tau \right)+E\left[n\left(t\right)-n\left(t-\tau \right)\right]$, where E[] is the mathematical expectation. Clearly, the noise term affects all symmetry indicators. An assessment of the impact of noise on the estimation of the various symmetry indicators will be necessary;
- Instead of comparing a signal x(t) with its transformed version Γ[x(t),τ], the concept of symmetrometry can be extended to **cross-symmetrometry** by comparing the reference signal x(t) and another signal y(t) transformed into $y^{\prime }\left(t\right)=\Gamma \left[y\left(t\right),\tau \right]$. The overall formula remains unchanged, only the measures $d_{j}\left(\tau \right)$ and $\rho_{j}\left(\tau \right)$ change:
  $$d_{j}\left(\tau \right)=\lim_{T\to \infty }\frac{1}{T}\int_{-T/2}^{T/2}\left|x\left(t\right)-\Gamma_{j}\left[y\left(t\right),\tau \right]\right|dt,$$
  $$\rho_{j}\left(\tau \right)=\lim_{T\to \infty }\frac{1}{T}\int_{-T/2}^{T/2}x\left(t\right)\times \Gamma_{j}\left[y\left(t\right),\tau \right]dt.$$

## 4. Results of the Study of Some Archetypal Signals

Now that we have all the necessary tools to generate and analyze symmetric signals, we propose the study of some signals that present specific characteristics.

### 4.1. Symmetry of Periodic Signals

#### 4.1.1. Symmetry of Periodic Signals from the Five Classes

Periodic signals are grouped into five different classes, and some periodic signals can be invariant under the four isometries simultaneously. Since there is an infinite number of generator patterns $x_{0}\left(t\right)$, there is, therefore, an infinite number of periodic signals. Here we will study only a few archetypal periodic signals. In Figure 4, signals from the five classes, based on the generator pattern $x_{0}\left(t\right)=Rect_{T/4}\left(t-T/8\right)\times sin\left(2\pi t/T\right)$ with a period of T=1 s, are represented. These signals of classes $P_{1}$, $P_{3}$, $P_{5}$, $P_{9}$, $P_{15}$ are represented over a time horizon of 4 s. To describe and compare these signals, we have calculated and reported in Table 4 the typed average symmetrometries $S_{j}$, the maxima of the symmetrometries $\sigma_{j}^{*}$, the symtaxis C, the symmetrometric value φ, the isorithm ϕ, the global symmetrometry S, and the symmentropy E.

As expected, class $P_{15}$ is the richest in diversity (E=1) and in the level of symmetrometry (S=0.72), while class $P_{1}$ is the least rich (E=0.46) and less symmetric (S=0.28).

It emerges from these calculations that the values of the symmetrometric index φ range between 0.39≤φ≤1 and increase as the class number C increases. The isorithm values range from 1≤ϕ≤4. It is verified that for class $P_{1}$, associated with a single invariance, the isorithm is ϕ=1, for classes $P_{3}$, $P_{5}$, $P_{9}$ associated with two invariances, the isorithm is ϕ=2, and for class $P_{15}$ associated with four invariances, the isorithm is ϕ=4. Furthermore, the isorithm ϕ clearly highlights the ratio between the number of periodic symbols (over the considered horizon) and the number of symmetry elements. For example, for the class $P_{15}$ signal, there are eight symmetry elements (four inversion centers and four mirror axes) and two patterns of period T=1 s. This results in a ratio of 8/2=4, which is equal to ϕ. This equality is verified for all classes. Finally, the values of the global symmetrometry range from 0.28≤S≤0.72, and those of the symmentropy from 0.46≤S≤1.00.

#### 4.1.2. Periodic Signals of Class $P_{15}$

**Figure 6:**
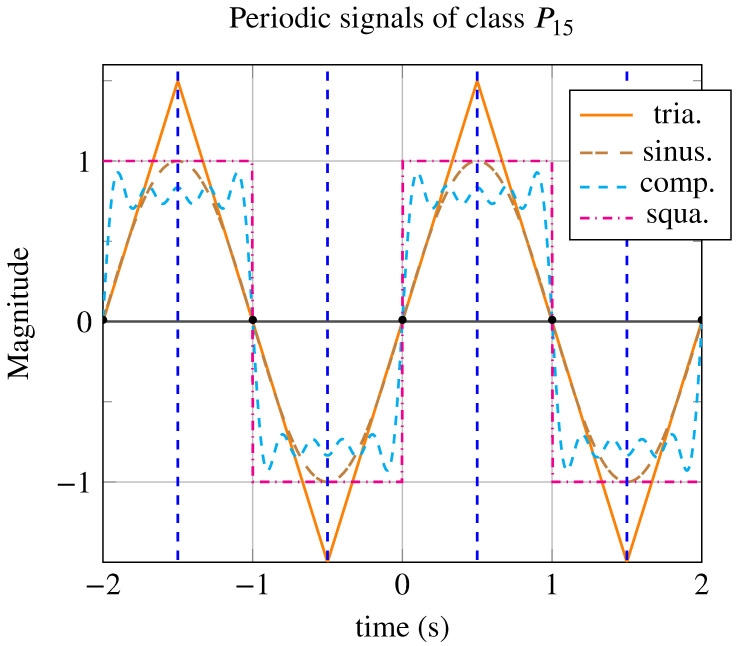
Periodic signals with a period of T=2 s from class $P_{15}$: triangular signal (in orange), sinusoidal signal (in brown), composite signal with 9 harmonic components (in cyan, dashed line), and square signal (in magenta, dash-dotted line). These signals possess the same symmetry elements and the same number, with only the generating patterns differing.

Since the most symmetric periodic signals are those of class $P_{15}$ compared to other classes, it seems appropriate to compare the signals of class $P_{15}$ with each other using different generating patterns. The various tested signals are shown in Figure 6, and their symmetry indicators are reported in Table 5.

From the indicators reported in Table 5, it appears that, although these signals have the same number of symmetry elements and the same number of periodic patterns, the square periodic signal has the highest indicators. This signal reaches the maximum global symmetrometry limit of S=1. The composite signal derived from the Fourier series decomposition of the square signal with nine harmonic components reaches a global symmetrometry of S=0.92. If the number of harmonic components tended toward infinity, its global symmetrometry would reach the maximum value.

It is notable that only the global symmetrometry distinguishes the three signals; the symmentropy, symmetrometry φ, symtaxis C, and isorithm ϕ are identical between them. On closer examination, we notice that the generating pattern of the square periodic signal possesses, in addition to the others, a mirror symmetry. This additional property is what places this signal at the top.

#### 4.1.3. Comparison of F-classification/P-classification

Firstly, having access to a **classification of periodic signals** is very important, especially if it is simple, and today, the F-classification, based on non-zero Fourier coefficients, is the only existing one. By adapting the calculation of the symtaxis C, it is possible, after calculating the various Fourier coefficients $a_{n}$ and $b_{n}$ (see definition in Equations (A1) and (A2), to estimate a symtaxis $C_{f}$ (see Appendix A.1) for the F-classification. However, this F-classification, compared to the P-classification, presents several disadvantages, the most important of which is that there are multiple classes for a signal exhibiting the same symmetry properties (surjection). Two examples are described below:

- As shown in Appendix A.1 in Table A1, with a well-chosen initial phase, seven classes of symmetric signals emerge from the F-classification, while only five classes appear in our P-classification. Classes $F_{4}$ and $F_{5}$ correspond to class $P_{9}$, and classes $F_{6}$ and $F_{7}$ correspond to class $P_{15}$.
- As shown in Table 6 and Table A1, for other initial phase values, other $F_{i}$ classes emerge while the $P_{i}$ classes remain unchanged. For example, for class $P_{15}$, a new class appears: $F_{5}$. For class $P_{5}$, two new classes appear: $F_{15}$ and $F_{6}$. Since the P-classification is invariant to the initial phase, it is therefore superior to the F-classification.

### 4.2. Symmetry of Periodic Signal with Duty Cycle

In several electrical engineering applications [17,18], the periodic signals used can become asymmetric, and for such applications, the duty cycle (The duty cycle α is defined as the ratio of the high state duration to the total period duration) α plays an important role.

To measure signal distortion, Total Harmonic Distortion (THD) is an important performance criterion for telecommunications systems [13,14], audio electronics [15,16], and electrical engineering [17,18], to name just a few. This distortion measure is based on harmonic power measurement and is defined as follows:

$$THD\left[x\left(t\right)\right]=\sqrt{\frac{\sum_{n=2}^{\infty }\left(a_{n}^{2}+b_{n}^{2}\right)}{a_{1}^{2}+b_{1}^{2}}},$$

where $a_{n}$ and $b_{n}$ are Fourier series coefficients defined in Equations (A1) and (A2) (in Appendix A.1).

An interesting case is that of the rectangular pulse train, where the duty cycle α is variable. Let us consider a generator signal $x_{0}\left(t\right)$ defined as follows:

$$x_{0}\left(t\right)=\left(Rect_{\alpha T}\left(t-\alpha T/2\right)-Rect_{(1-\alpha )T}\left(t-\left(2-\alpha \right)T/2\right)\right).$$

**Figure 7:**
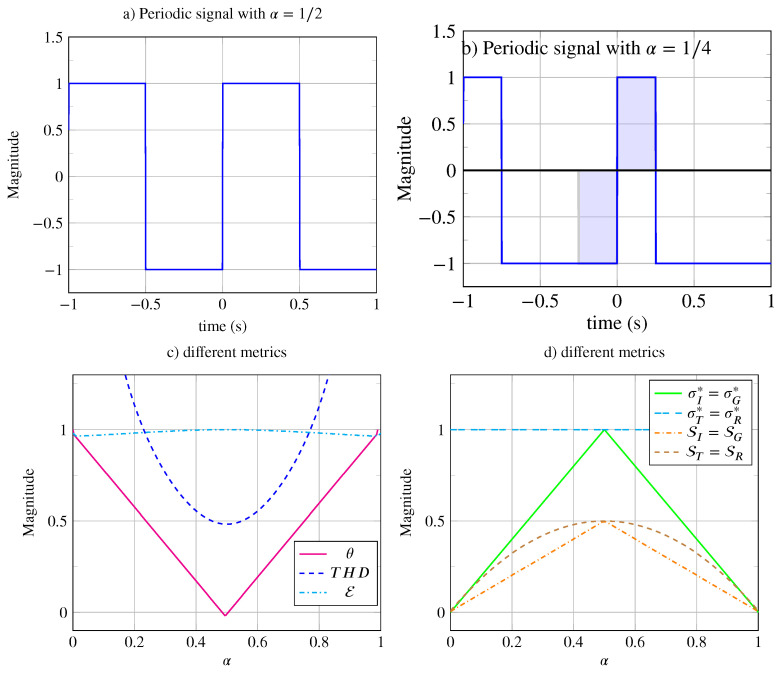
Periodic signals with variable duty cycle α. (**a**) Signal with a duty cycle of α=1/2 and infinite odd symmetry range. (**b**) Signal with a duty cycle of α=1/4, highlighting the limited range (T/4 on either side of the inversion center at t=0) of the signal’s odd symmetry (area colored in blue). (**c**) Various metrics as a function of the duty cycle: THD(α) (dashed line), E(α) (dash-dotted line), and θ(α). (**d**) Different metrics $S_{T}\left(\alpha \right)=S_{R}\left(\alpha \right)$ (dashed brown line), $S_{I}\left(\alpha \right)=S_{G}\left(\alpha \right)$ (dash-dotted orange line), $\sigma_{T}^{*}\left(\alpha \right)=\sigma_{R}^{*}\left(\alpha \right)$ (dashed cyan line), and $\sigma_{I}^{*}\left(\alpha \right)=\sigma_{G}^{*}\left(\alpha \right)$ (solid green line) as a function of the duty cycle.

The square periodic signal generated with α=1/2 belongs to class $P_{15}$, as shown in Figure 7a, where two types of symmetry elements can be distinguished: reflection axes of infinite range $L_{x}\left(kT\right)=L_{x}\left(T/2+kT\right)=\infty$ and inversion centers of infinite range $L_{x}\left(\pm T/4+kT\right)=\infty$.

With α=1/4, the signal belongs to class $P_{3}$, as shown in Figure 7b. Indeed, this signal remains invariant under all translations of kT and all vertical reflections located at ±T/8+kT. However, this signal is no longer invariant under glide reflection or inversion. The signal moves from class $P_{15}$ to class $P_{3}$. Moreover, although its inversion invariance is no longer global, it still exists but its range is limited, making inversion symmetry local. Here, the range of inversion symmetry is limited to $L_{x}\left(kT\right)=L_{x}\left(kT+T/4\right)=T/4$. In Figure 7b, the range of inversion symmetry at t=0 is represented by the blue-colored areas.

For the rectangular pulse train with a variable duty cycle, it is theoretically possible to calculate the THD (see [14]):

$$THD\left[x\left(t\right),\alpha \right]=\sqrt{\frac{\alpha \left(1-\alpha \right)\pi^{2}}{2\sin{\left(\alpha \pi \right)}^{2}}-1},$$

the same applies to the distortion indicator expressed as follows:

θ[α]=|2α−1|.

This is shown in Figure 7c. For α=1/2, THD≈1/2, and it flattens significantly around α=1/2. When the duty cycle approaches its extreme values, it becomes undefined and increases rapidly. As α→0, the signal tends toward a Dirac comb, and $\lim_{\alpha \to 0}THD\left[x\left(t\right),\alpha \right]=\infty$. When α→1, the signal tends toward a unit constant, and $\lim_{\alpha \to 1}THD\left[x\left(t\right),\alpha \right]=\infty$. Due to its shape, this function does not seem well-suited for describing the distortion level, which increases too quickly at the extremes and is too flat around α=1/2. Furthermore, the THD indicator should be zero when there is no distortion, i.e., for α=1/2, whereas THD[x(t),1/2]≈1/2. Our indicator, on the other hand, displays a zero value when there is no distortion θ[1/2]=0 (see Figure 7c). The distortion indicator θ[α] is linear and finite for its extreme values. As the distortion decreases (i.e., as α increases from 0 to 0.5), the indicator decreases linearly to zero. The symmentropy E[α] varies very little and remains close to unity; for this application, this indicator does not seem relevant.

As for the average typed symmetrometries, they are shown in Figure 7d as a function of the duty cycle α. The average translation and vertical reflection symmetrometries are identical $S_{T}=S_{R}$ and maximal for $S_{T}^{*}\left(1/2\right)=S_{R}^{*}\left(1/2\right)=1/2$. The average inversion and glide reflection symmetrometries are identical $S_{I}=S_{G}$ and maximal for $S_{I}^{*}\left(1/2\right)=S_{G}^{*}\left(1/2\right)=1/2$. The variable duty cycle signal is therefore invariant under translation and vertical reflection; it is no longer invariant under inversion and glide reflection except for α=1/2. The maximum typed translation and reflection symmetrometries are identical $\sigma_{T}^{*}=\sigma_{R}^{*}=1$ and are shown in Figure 7d. The maximum typed inversion and glide reflection symmetrometries are identical $\sigma_{I}^{*}=\sigma_{G}^{*}$, they have a triangular shape and are maximal at α=1/2.

### 4.3. Shock Waves

Other applications, in which periodic signals can transition from one class to another, involve the study of mechanical wave propagation in nonlinear media [19,20]. As the waves propagate, they deform and may lose certain symmetries. This deformation is related to the nonlinear nature of the medium through which the wave propagates and is often measured using the B/A indicator. This indicator, defined as follows,

$$B/A=\frac{\sqrt{a_{2}^{2}+b_{2}^{2}}}{\sqrt{a_{1}^{2}+b_{1}^{2}}},$$

is preferred over THD because the frequency band of the sensors is too limited to capture higher harmonics. Note that the Fourier coefficients $a_{n}$ and $b_{n}$ are defined in Equations (A1) and (A2) (in Appendix A.1).

**Figure 8:**
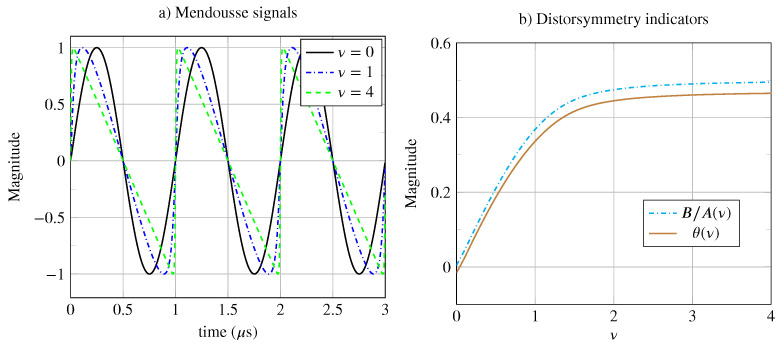
(**a**) Mendousse signal for different values of the shock wave coefficient ν. (**b**) Distortion indicator B/A(ν) and distortion symmetry indicator θ(ν).

In the example shown in Figure 8 and based on the work of Mendousse [23], a mono-frequency plane wave propagates through a thermo-viscous nonlinear medium. Numerical solutions can be obtained through simulations with the simulator “k-wave” (http://www.k-wave.org/documentation/example_na_modelling_nonlinearity.php; https://fr.mathworks.com/matlabcentral/fileexchange/120178-k-wave/, accessed on 26 October 2024). Depending on the value of the shock parameter ν, the higher the value of ν, the more distorted the wave becomes. In Figure 8a, three values of the parameter ν are proposed. For ν=0, there is no distortion, and the signal is of class $P_{15}$ because it is invariant under the four isometries. For ν=1, the wave distortion is moderate, and for ν=4, the distortion is significant. As ν→∞, the signal becomes of class $P_{5}$. The signal is globally asymmetrical since only the invariances by translation and inversion are preserved.

Among the proposed indicators, our distortion indicator θ measures the asymmetries linked to the shock wave distortion, and B/A measures the second-order harmonic distortion [19,20]. From Figure 8b, the distortion symmetry θ[ν] is very similar to the coefficient B/A. As ν→∞, the indicator B/A→1/2 and the distortion symmetry θ→1/2.

In summary, the two proposed indicators have sufficient dynamics to help characterize the shock wave, especially in the range 0≤ν≤2. A measurement of the harmonic indicator B/A or the distorsymmetry θ could allow one to estimate the shock wave coefficient ν associated with the mechanical parameters of the explored medium. This example simply shows that there are at least two ways of obtaining similar results with two very different methods.

### 4.4. Symmetry of Non-Periodic Symmetric Signals

Although periodic signals represent the majority of symmetrical signals, non-periodic signals are no less interesting. As mentioned previously, these non-periodic symmetrical signals no longer exhibit invariance by translation or glide reflection; only vertical reflection, inversion, or a combination of the two exist.

**Figure 9:**
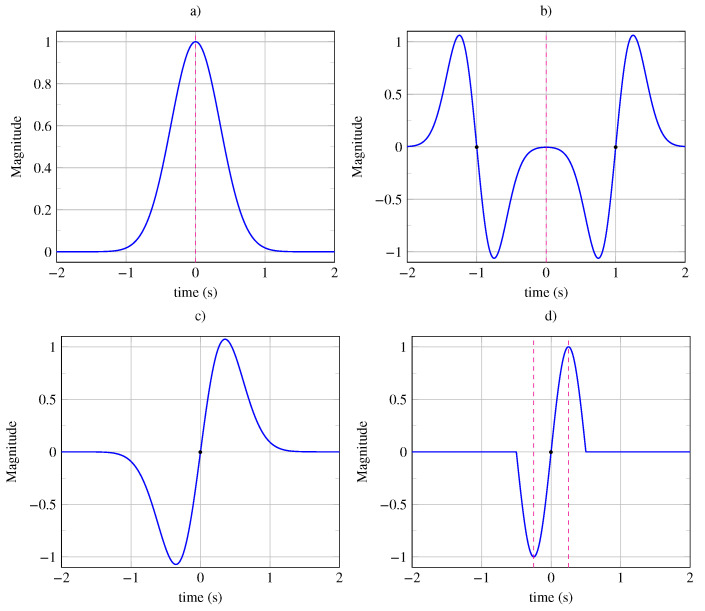
Non-periodic symmetrical signals: (**a**) Signal exhibiting a global mirror symmetry (invariance under vertical reflection) with a vertical reflection axis at t=0. (**b**) Signal exhibiting a global mirror symmetry (invariance under vertical reflection) with a vertical reflection axis at t=0 and local symmetries (with limited range) under inversion with two inversion centers at t=−1 s and t=1 s. (**c**) Signal exhibiting a global central symmetry (invariance under inversion) with an inversion center at t=0. (**d**) Signal exhibiting a global central symmetry (invariance under inversion) with an inversion center at t=0 and local mirror symmetries (with limited range) with two mirror axes at t=−1/4 s and t=1/4 s.

We propose to study archetypes of non-periodic symmetrical signals, represented in Figure 9. The signals shown in Figure 9a,c exhibit only global invariances. The signals in Figure 9b,d exhibit both global and local symmetries.

The signal $x_{r}\left(t\right)=e^{-at^{2}}$ shown in Figure 9a exhibits an invariance under mirror reflection, with its reflection axis at t=0. The signal $x_{i}\left(t\right)=-2t\times e^{-at^{2}}$ shown in Figure 9c exhibits an invariance under inversion, with its inversion center at t=0. The signal $x_{ir}\left(t\right)=e^{-a{\left(t-1\right)}^{2}}-e^{-a{\left(t+1\right)}^{2}}$ shown in Figure 9b exhibits an invariance under mirror reflection at t=0 and local symmetries around inversion centers at t=±1 s, with the range being limited to L(±1)=1 s. The signal $x_{ri}\left(t\right)=\sin\left(2\pi t/T\right)\times Rect_{T}\left(t\right)$ with a period of T=1 s, shown in Figure 9b, exhibits an inversion symmetry around its center at t=0 and local symmetries around reflection axes at t=±1/4 s, with the range being limited to L(±1/4)=1/4 s.

The symmetry indicators are reported in Table 7. The signals $x_{ir}\left(t\right)$ and $x_{ri}\left(t\right)$ exhibit similar results and show identical symmetric score φ=0.38, even though they do not belong to the same class. The global symmetric score is higher for the signals $x_{r}\left(t\right)$ and $x_{i}\left(t\right)$, which do not exhibit additional local symmetries. It can be observed that global symmetry is obtained with $\sigma_{R}^{*}=1$ or $\sigma_{I}^{*}=1$ and a local symmetry with $\sigma_{R}^{*}=1/2$ or $\sigma_{I}^{*}=1/2$. It can also be verified that the symtaxis correctly estimates the appropriate class of membership.

## 5. Discussion and Conclusions

In our article, we adopted the mathematical framework of group theory (specifically the frieze group) to study both periodic and non-periodic signals. Using this framework, we proposed two new classifications, the P-classification and the Q-classification, as complements to the F-classification. After adjusting the phase at the origin of the signals, the F-classification encompasses seven classes, while the P-classification includes only five. By comparing these classifications, we found that P-classification is more general and bijective, meaning each signal type uniquely corresponds to one class, unlike F-classification.

Additionally, we introduced the symtaxis indicator C, which allows for the direct estimation of a signal’s classification based solely on its data without requiring knowledge of its expression. Notably, C is invariant to changes in the signal’s phase, unlike the estimation of classes $C_{f}$ (see Equation (A3)) derived from Fourier series coefficients.

In the Q-classification for symmetric non-periodic signals, only improper isometry invariances were found, which reduced global symmetry descriptions to just two classes. It is important to remember that the F, P, and Q classifications address global symmetries, i.e., symmetries tied to global invariances. By introducing new indicators such as the maxima of typed symmetrometries $\sigma_{j}^{*}$, we were able to highlight local symmetries within limited domains.

For example, the analysis of a periodic square wave signal (with α=1/2), illustrated in Figure 7a, showed that the maxima of the typed symmetrometries were identical, with $\sigma_{T}^{*}=\sigma_{R}^{*}=\sigma_{I}^{*}=\sigma_{G}^{*}=1$, indicating global symmetry. With α=1/4, the maxima of the typed symmetrometries were $\sigma_{T}^{*}=\sigma_{R}^{*}=1$ (global symmetry) and $\sigma_{I}^{*}=\sigma_{G}^{*}=1/2$ (local symmetry), where the extent of the inversion centers was 1/4 of the period, while the reflection axes extended infinitely. A similar analysis applies to non-periodic signals, such as the one depicted in Figure 9, which exhibits both global even symmetry and local odd symmetry. In this case, the inversion center’s extent was 1 s, with the maxima of typed symmetrometries being $\sigma_{R}^{*}=1$ and $\sigma_{I}^{*}=1/2$. Therefore, the maxima of typed symmetrometries serve as valuable indicators for determining whether the symmetry is global ($\sigma_{j}^{*}=1$) or local ($\sigma_{j}^{*}=1/2$).

Building on this mathematical framework, we also proposed an iterative algorithm (see Equation (1)) to generate symmetric signals from an initial generating pattern $x_{0}\left(t\right)$, a chosen number of iterations *n*, and isometries selected from the four available options. In telecommunications applications, knowing the sequence of isometries in advance could provide an opportunity to encode this sequence securely, enhancing message detection.

To quantify and qualify the level of symmetry present in periodic signals, we introduced the concepts of typed symmetrometries Sj, global symmetrometries S, symmetrometries φ, distorsymmetry θ, and symmentropy E, applying them to the study of various signals. Among these indicators, the symtaxis indicator C and the isorithm ϕ are better suited for classifying periodic signals. For assessing symmetry levels, the symmetrometry indicators S, φ, and symmentropy E are more appropriate as their values increase with richer symmetry. For instance, among five signal types classified under P-classification (see Table 4), the signal with only one invariance (class $P_{1}$) had the lowest indicators, while the signal with the most invariances (class $P_{15}$) had the highest. This result supports the conclusion that class $P_{15}$ signals, which are invariant under all four isometries, exhibit the highest global symmetrometry and symmentropy.

Even though all periodic signals in class $P_{15}$ share the same number and positions of inversion centers and reflection axes, the square wave signal has the highest indicators compared to others, reaching their maximum value. This can be attributed to the fact that, unlike sinusoidal and triangular signals, the square wave’s generating pattern is already symmetric.

Finally, when studying signals with varying duty cycles α or shock wave coefficients ν, we found that certain symmetries could be disrupted to create global dissymmetry or even asymmetry. To illustrate this, we showed that the infinite extent of symmetry in some signals (around an inversion center or reflection axis) could be limited depending on the duty cycle. We demonstrated that the distortion caused by duty cycles α=0 and α=1 was better measured using our new distortion indicator θ rather than Total Harmonic Distortion (THD). Unlike THD, which is undefined for extreme values, distortion symmetry θ is well-defined for these cases. Additionally, we showed that THD evolves too quickly for small α values and too slowly for α=1/2. In our study of shock waves, we found that distortion symmetry θ closely matches the harmonic indicator B/A. For this application, both distortion indicators yielded similar results, with distortion symmetry offering the advantage of being independent of signal power, as it only responds to shape changes involving dissymmetry.

Looking ahead, many possibilities for future work exist, though we will mention only a few here. Our study was deliberately limited to deterministic signals; clearly, if the signals were corrupted by noise, the indicators would be affected. However, using a metric based on the correlation coefficient, as discussed in Section 3.5, should yield results less sensitive to noise.

For research on asymmetry in stochastic signals through time irreversibility, the statistical tools proposed in our work were limited to one isometry (vertical reflection). It would be interesting to extend these tools to account for other isometries.

Additionally, while this work focused on isometry-based symmetries, other types of symmetry, such as conformal symmetries (invariance under scaling), deserve further investigation, particularly for the study of fractal signals.

Lastly, since symmetry-based distortion indicators outperform traditional measures like THD for certain signals, applying these indicators to other fields would be a wise direction for future research.

## Figures and Tables

**Table 1:** **Cayley table** illustrating how the composition of any two isometries operating on the signal x(t) modifies it. The composition of 2 isometries is an isometry: $\Gamma_{T}\left[x\left(t\right),b\right]\;\circ \;\Gamma_{T}\left[x\left(t\right),a\right]=x\left(t-\left(b+a\right)\right)$, $\Gamma_{T}\left[x\left(t\right),a\right]\;\circ \;\Gamma_{T}\left[x\left(t\right),-a\right]=\Gamma_{T}\left[x\left(t\right),0\right]=x\left(t\right)=\Gamma_{\epsilon }\left[x\left(t\right)\right]$ where $\Gamma_{\epsilon }$ is the identity operation. The values a,b,c,d,e,f,g,h∈N represent the positions where the isometry operates. Details are presented in Appendix A.4.

→∘→	$\Gamma_{T}\left[x\left(t\right),a\right]$	$\Gamma_{R}\left[x\left(t\right),e\right]$	$\Gamma_{I}\left[x\left(t\right),g\right]$	$\Gamma_{G}\left[x\left(t\right),c\right]$
$\Gamma_{T}\left[x\left(t\right),b\right]$	x(t−(b+a))	x(−t+2e−b)	−x(−t+2g−b)	−x(t−c−b)
$\Gamma_{R}\left[x\left(t\right),f\right]$	x(−t+a+2f)	x(t−2e+2f)	−x(t−2g+2f)	−x(−t+c+2f)
$\Gamma_{I}\left[x\left(t\right),h\right]$	−x(−t+a+2h)	−x(t−2e+2h)	x(t−2g+2h)	x(−t+c+2h)
$\Gamma_{G}\left[x\left(t\right),d\right]$	−x(t−a−d)	−x(−t+2e−d)	x(−t+2g−2d)	x(t−c−d)

**Table 2:** The classification of global symmetry for periodic (P-Classification ) and non-periodic (Q-Classification) signals is based on the type, number of iterations, group, and isometry used. There are five possible types of periodic signals and two types of non-periodic signals. In the case of periodic signals, the number of iterations is infinite, while it is limited to two for non-periodic symmetric signals. The fourth line indicates the generating isometries. The signals of class $P_{1}$ and $P_{9}$ are associated with the infinite cyclic group $C_{\infty }$, with a single type of generating isometry. The signals of class $P_{3}$, $P_{5}$ and $P_{15}$ are associated with the infinite dihedral group $D_{\infty }$, with two generating isometries. The signals of class $Q_{2}$ and $Q_{4}$ are associated with the dihedral group $D_{2}$. The composition G∘R is identical to I∘R. The last line of the table indicates the various isometries for which the signals are invariant.

Type	$P_{1}$	$P_{3}$	$P_{5}$	$P_{9}$	$P_{15}$	$Q_{2}$	$Q_{4}$
Nb of iter.	*∞*	*∞*	*∞*	*∞*	*∞*	2	2
Group	$C_{\infty }$	$D_{\infty }$	$D_{\infty }$	$C_{\infty }$	$D_{\infty }$	$D_{2}$	$D_{2}$
Group generator	T	T, R	T, I	G	I, G ∘ R	R	I
Invariance	T	T, R	T, I	T, G	T, R, I, G	R	I

**Table 3:** Table providing a binary coding scheme for the classification of symmetric signals based on the isometries (Translation T, Vertical Reflection R, Inversion I, and Glide Reflection G). Signals are divided into the P-classification for periodic signals and the Q-classification for non-periodic symmetric signals.

G	I	R	T	Code	Class
**($2^{3}$)**	**($2^{2}$)**	**($2^{1}$)**	**($2^{0}$)**		
0	0	0	1	1	$P_{1}$
0	0	1	1	3	$P_{3}$
0	1	0	1	5	$P_{5}$
1	0	0	1	9	$P_{9}$
1	1	1	1	15	$P_{15}$
0	0	1	0	2	$Q_{2}$
0	1	0	0	4	$Q_{4}$

**Table 4:** Indicators of the level of symmetry for symmetric signals of P-classification: There are five different classes: $P_{1}$, $P_{3}$, $P_{5}$, $P_{9}$, and $P_{15}$. The integer parts are in bold. The signal from class $P_{15}$ has the highest level of symmetry. The symmetrometry φ, the global symmetrometry S, and the symmentropy E increase as the class number increases.

Signal	P-Class.	$S_{T}$	$S_{R}$	$S_{I}$	$S_{G}$	$\sigma_{T}^{*}$	$\sigma_{R}^{*}$	$\sigma_{I}^{*}$	$\sigma_{G}^{*}$	C	φ	ϕ	S	E
$x_{t}\left(t\right)$	$P_{1}$	0.36	0.36	0.00	0.00	**1**.00	**0**.54	**0**.00	**0**.00	1	0.39	1	0.28	0.46
$x_{r}\left(t\right)$	$P_{3}$	0.36	0.36	0.00	0.00	**1**.00	**1**.00	**0**.00	**0**.00	3	0.50	2	0.36	0.50
$x_{i}\left(t\right)$	$P_{5}$	0.36	0.36	0.36	0.36	**1**.00	**0**.59	**1**.00	**0**.59	5	0.80	2	0.57	0.98
$x_{g}\left(t\right)$	$P_{9}$	0.36	0.36	0.36	0.36	**1**.00	**0**.74	**0**.74	**1**.00	7	0.87	2	0.63	0.99
$x_{ir}\left(t\right)$	$P_{15}$	0.36	0.36	0.36	0.36	**1**.00	**1**.00	**1**.00	**1**.00	15	1.00	4	0.72	1.00

**Table 5:** Comparison of four periodic signals from class $P_{15}$: square, composite (Fourier series decomposition of the square signal with nine components), sinusoidal, and triangular. Of all four signals, only the symmetrometry allows differentiation. The four signals exhibit maximum symmentropy E=1 due to equiprobability. The signal with the highest symmetrometry is the square signal with S=1, followed by the composite signal with nine components at S=0.92, the sinusoidal signal with S=0.72, and the triangular signal with S=0.66.

Signals	Class. P	$S_{T}$	$S_{R}$	$S_{I}$	$S_{G}$	C	φ	ϕ	S	E
square	$P_{15}$	0.5	0.5	0.5	0.5	15	1	4	1	1
comp	$P_{15}$	0.46	0.46	0.46	0.46	15	1	4	0.92	1
sinus	$P_{15}$	0.36	0.36	0.36	0.36	15	1	4	0.72	1
triangle	$P_{15}$	0.33	0.33	0.33	0.33	15	1	4	0.66	1

**Table 6:** Comparison of F-classification and P-classification. The signal $x_{sw}\left(t\right)=\arctan\left(\tan\left(2\pi t/T+\psi \right)\right)$ belongs to class $P_{15}$ and the signal $x_{tr}\left(t\right)=\arcsin\left(\sin\left(2\pi t/T+\psi \right)\right)$ belongs to class $P_{5}$. For each of these two signals, there are three different classes in the F-classification.

Signal	ψ	$a_{2n}$	$a_{2n-1}$	$b_{2n}$	$b_{2n-1}$	F-Class.	P-Class.	φ	ϕ	S
$x_{tr}\left(t\right)$	0	0	0	0	≠0	$F_{1}$	$P_{15}$	1	4	0.67
	π/4	0	≠0	0	≠0	$F_{5}$				
	π/2	0	≠0	0	0	$F_{4}$				
$x_{sw}\left(t\right)$	0	0	0	≠0	≠0	$F_{3}$	$P_{5}$	0.76	2	0.51
	π/4	≠0	≠0	≠0	≠0	$F_{15}$				
	π/2	0	≠0	≠0	0	$F_{6}$				

**Table 7:** Indicators for the following non-periodic signals: $x_{1}\left(t\right)=e^{-at^{2}}$ signal exhibiting a global mirror symmetry, $x_{2}\left(t\right)=e^{-a{\left(t-1\right)}^{2}}-e^{-a{\left(t+1\right)}^{2}}$ signal exhibiting a global mirror symmetry and local central symmetries, $x_{3}\left(t\right)=-2te^{-t^{2}}$ signal exhibiting a global central symmetry, and $x_{4}\left(t\right)=\sin\left(\frac{2\pi }{T}t\right)\times Rect_{T}\left(t\right)$ signal exhibiting a global central symmetry and local mirror symmetries. Global symmetry is observed from $\sigma_{R}^{*}=1$ or $\sigma_{I}^{*}=1$ while local symmetry is observed from $\sigma_{R}^{*}=1/2$ or $\sigma_{I}^{*}=1/2$.

Signal	Class. Q	$S_{T}$	$S_{R}$	$S_{I}$	$S_{G}$	$\sigma_{T}^{*}$	$\sigma_{R}^{*}$	$\sigma_{I}^{*}$	$\sigma_{G}^{*}$	C	φ	S	E
$x_{r}\left(t\right)$	$Q_{2}$	0.26	0.26	0.00	0.00	**1**.00	**1**.00	**0**.00	**0**.00	2	0.25	0.13	0.00
$x_{ir}\left(t\right)$	$Q_{2}$	0.08	0.08	0.08	0.08	**1**.00	**1**.00	**0**.50	**0**.50	2	0.38	0.06	0.46
$x_{i}\left(t\right)$	$Q_{4}$	0.17	0.17	0.17	0.17	**1**.00	**0**.44	**1**.00	**0**.44	4	0.36	0.12	0.42
$x_{ri}\left(t\right)$	$Q_{4}$	0.06	0.06	0.06	0.06	**1**.00	**0**.50	**1**.00	**0**.50	4	0.38	0.05	0.46

**Figure A1:**
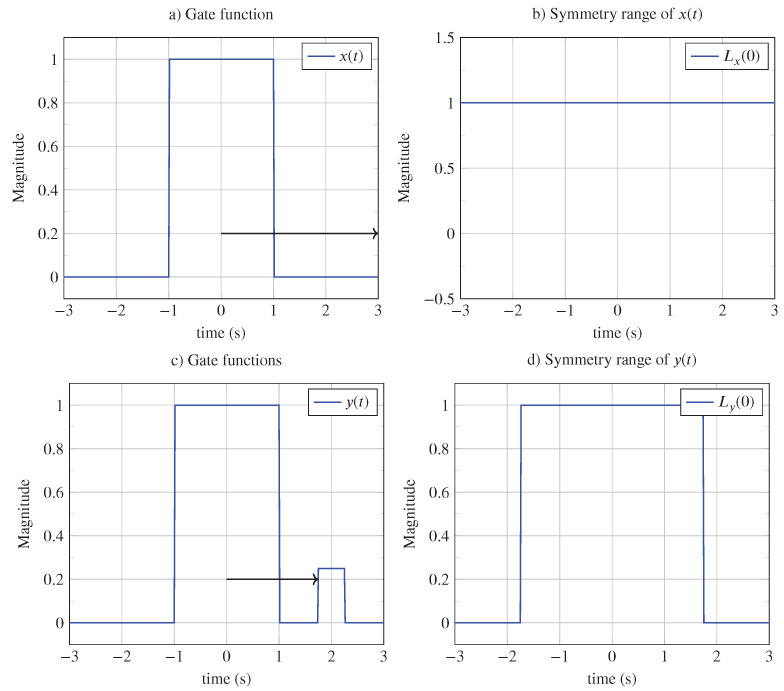
Measurement of the range of the mirror axis at $t_{0}=0$ s: (**a**) Rectangular function centered at t=0 s. (**b**) Infinite range of the mirror axis at t=0 s. (**c**) Distorted rectangular function centered at t=0. (**d**) Range limited to 1.75 s from the mirror axis at t=0.

## Data Availability

The original contributions presented in the study are included in the article, further inquiries can be directed to the corresponding author.

## Appendix A

### Appendix A.1. Symmetry Classification and Fourier Series

There is an approach to classifying symmetric periodic signals by decomposing them into a Fourier series [24]:

$$x\left(t\right)=\frac{a_{0}}{2}+\sum_{n=1}^{\infty }a_{n}\cos\left(2\pi nf_{0}t\right)+b_{n}\sin\left(2\pi nf_{0}t\right),$$

with $f_{0}=1/T$, $a_{0}=\frac{1}{T}\int_{t_{0}}^{t_{0}+T}x\left(\tau \right)d\tau$,

(A1) $$a_{n}=\frac{2}{T}\int_{t_{0}}^{t_{0}+T}x\left(\tau \right)\cos\left(2\pi nf_{0}\tau \right)d\tau$$

and with

(A2) $$b_{n}=\frac{2}{T}\int_{t_{0}}^{t_{0}+T}x\left(\tau \right)\sin\left(2\pi nf_{0}\tau \right)d\tau$$

This classification, which we called F-classification, links the coefficients $a_{n}$ and $b_{n}$ based on a specific type of symmetry, with $a_{2n}$ and $b_{2n}$ representing even coefficients and $a_{2n-1}$ and $b_{2n-1}$ representing odd coefficients.

In Table A1, seven signals are considered. The first signal (line 2 of Table A1) is an arbitrary periodic signal with $a_{n}\neq 0$ and $b_{n}\neq 0$. The second signal (line 3 of Table A1) is an even periodic signal x(−t)=x(t). The third signal (line 4 of Table A1) is an odd periodic signal x(−t)=−x(t). The fourth signal (line 5 of Table A1) is an even half-wave periodic signal verifying x(t+T/2)=x(t) and x(−t)=x(t), with the period divided into two symmetric patterns. The fifth signal (line 6 of Table A1) is an odd half-wave periodic signal verifying x(t+T/2)=x(t) and x(−t)=−x(t), with the period divided into two asymmetric patterns.

The sixth signal (line 7 of Table A1) is an even quarter-wave periodic signal. A quarter-wave signal is also a half-wave signal, where the half-period is divided into two asymmetric patterns. The seventh signal (line 8 of Table A1) is an odd quarter-wave periodic signal. A quarter-wave signal is also a half-wave signal, where the half-period is divided into two symmetric patterns.

Similar to the symtaxis indicator C, we propose a Fourier symtaxis indicator $C_{f}$ calculated directly from the estimation of the Fourier series coefficients:

(A3) $$C_{f}=2^{0}u\left[b_{2n-1}\right]+2^{1}u\left[b_{2n}\right]+2^{2}u\left[a_{2n-1}\right]+2^{3}u\left[a_{2n}\right],$$

where u[] is the Heaviside function defined as u[x(t)≤0]=0 and u[x(t)>0]=1.

**Remark** **A1.**

- *The signals considered in Table A1 have an initial phase that guarantees either even or odd symmetry. For other initial phase values, other configurations may arise (see Table 6 in Section 4.1.3);*
- *The Fourier series coefficients can be obtained either by analytical calculation when the signal’s expression is known or by numerical calculation if the expression is unknown but numerical values are available.*

**Table A1:** Classifications F and P. Table of Fourier coefficients for seven types of periodic signals ∀n integer: arbitrary signal, even signal, odd signal, 1/2 even and odd wave signals, 1/4 even and odd wave signals. P-Classification from Section 2.4. $a_{0}$: DC component, $a_{2n}$ and $b_{2n}$: even coefficients, $a_{2n-1}$ and $b_{2n-1}$: odd coefficients. There is a single F-classification for all signals except for the P-classification, for which half-wave and quarter-wave signals have the same class.

Signal /Coef.	F-Class.	$C_{f}$	$a_{0}$	$a_{2n}$	$a_{2n-1}$	$b_{2n}$	$b_{2n-1}$	P-Class.
arbitrary	$F_{15}$	15	-	≠0	≠0	≠0	≠0	$P_{1}$
even	$F_{12}$	12	-	≠0	≠0	0	0	$P_{3}$
odd	$F_{3}$	3	0	0	0	≠0	≠0	$P_{5}$
1/2 wave even	$F_{5}$	5	0	0	≠0	0	≠0	$P_{9}$
1/2 wave odd	$F_{10}$	10	0	≠0	0	≠0	0	$P_{9}$
1/4 wave even	$F_{4}$	4	0	0	≠0	0	0	$P_{15}$
1/4 wave odd	$F_{1}$	1	0	0	0	0	≠0	$P_{15}$

### Appendix A.2. Symmetry Elements and Range

The symmetry elements are positions around which two equidistant points are either equal (reflection axis) or opposite (center of inversion). The maximum distance separating these two equal or opposite points from the signal $x(t_{0})$ at position $t_{0}$ gives the range of the symmetry $L_{x}\left(t_{0}\right)$, which is defined as follows:

$$L_{x}\left(t_{0}\right)=\frac{1}{2}\int L_{x}\left(t_{0},\tau \right)d\tau ,$$

where $L_{x}\left(t_{0},\tau \right)=u(|x\left(t_{0}\right)-\Gamma_{j}\left[x\left(t\right),\tau \right]\left|\leq \iota \right)$, u(:) is the Heaviside function, and ι is a very small value (e.g., ι=0.01). The function $L_{x}\left(t_{0},\tau \right)=1$ when the inequality $|x\left(t_{0}\right)-\Gamma_{j}\left[x\left(t\right),\tau \right]|\leq \iota$ is satisfied; otherwise, $L_{x}\left(t_{0},\tau \right)=0$.

**Example** **A1.**
*The rectangular function $x\left(t\right)=Rect_{T}\left(t\right)$ of duration T=2 s, having a symmetry axis at t=0, is shown in Figure A1a. The range of symmetry is Lx(0)=∞ (see Figure A1b). However, when the rectangular function is disturbed by another rectangular function defined as $y\left(t\right)=RectT\left(t\right)+\frac{1}{4}Rect_{T/4}\left(t-2\right)$, located at t=2 s and with a width of 0.5 s (see Figure A1c), the symmetry is broken. The range of symmetry at position t=0 is then limited to $L_{y}\left(0\right)=1.75$ s on either side of the ordinate axis (see Figure A1c).*

### Appendix A.3. Specific Cayley Tables

**Table A2:** **Cayley table of the compositions of isometries for friezes**. The symmetry group of the periodic signals is a subgroup of the symmetry group of friezes, and it is included within the latter (non-colored part).

−∘−	$\Gamma_{T}\left[x\left(t\right),a\right]$	$\Gamma_{R}\left[x\left(t\right),e\right]$	$\Gamma_{I}\left[x\left(t\right),g\right]$	$\Gamma_{G}\left[x\left(t\right),c\right]$	$\Gamma_{H}\left[x\left(t\right)\right]$
ine $\Gamma_{T}\left[x\left(t\right),b\right]$	$\Gamma_{T}\left[x\left(t\right),-\left(b+a\right)\right]$	$\Gamma_{R}\left[x\left(t\right),-b+2e\right]$	$\Gamma_{I}\left[x\left(t\right),-b+2g\right]$	$\Gamma_{G}\left[x\left(t\right),-\left(b+c\right)\right]$	$\Gamma_{G}\left[x\left(t\right),b\right]$
ine $\Gamma_{R}\left[x\left(t\right),f\right]$	$\Gamma_{R}\left[x\left(t\right),2f+a\right]$	$\Gamma_{T}\left[x\left(t\right),2f-2e\right]$	$\Gamma_{G}\left[x\left(t\right),2f-2g\right]$	$\Gamma_{I}\left[x\left(t\right),c+2f\right]$	$\Gamma_{I}\left[x\left(t\right),f\right]$
ine $\Gamma_{I}\left[x\left(t\right),h\right]$	$\Gamma_{I}\left[x\left(t\right),2h+a\right]$	$\Gamma_{G}\left[x\left(t\right),2h-2e\right]$	$\Gamma_{T}\left[x\left(t\right),2h-2g\right]$	$\Gamma_{R}\left[x\left(t\right),2h+c\right]$	$\Gamma_{R}\left[x\left(t\right),h\right]$
ine $\Gamma_{G}\left[x\left(t\right),d\right]$	$\Gamma_{G}\left[x\left(t\right),-\left(d+a\right)\right]$	$\Gamma_{I}\left[x\left(t\right),2e-2d\right]$	$\Gamma_{R}\left[x\left(t\right),2g-2d\right]$	$\Gamma_{T}\left[x\left(t\right),-\left(d+c\right)\right]$	$\Gamma_{T}\left[x\left(t\right),d\right]$
ine $\Gamma_{H}\left[x\left(t\right)\right]$	$\Gamma_{G}\left[x\left(t\right),a\right]$	$\Gamma_{I}\left[x\left(t\right),c\right]$	$\Gamma_{R}\left[x\left(t\right),e\right]$	$\Gamma_{T}\left[x\left(t\right),g\right]$	ϵ

**Table A3:** **Non-commutative Cayley table** for a pairwise symmetry-generating pattern (∩) of duration *T* and centered at 0. +A=∩∩, −A=∪∪, +B=∩∪, and −B=∪∩.

−∘−	$\Gamma_{T}\left[x\left(t\right),T\right]$	$\Gamma_{R}\left[x\left(t\right),T/2\right]$	$\Gamma_{I}\left[x\left(t\right),T/2\right]$	$\Gamma_{G}\left[x\left(t\right),T\right]$
$\Gamma_{T}\left[x\left(t\right),0\right]$	+A	+A	+B	+B
$\Gamma_{G}\left[x\left(t\right),0\right]$	−A	−A	−B	−B
$\Gamma_{R}\left[x\left(t\right),T/2\right]$	−A	−A	−B	−B
$\Gamma_{I}\left[x\left(t\right),T/2\right]$	+A	+A	+B	+B

**Table A4:** **Non-commutative Cayley table** for an odd symmetry-generating pattern (∩∪) of duration *T* and centered at 0. +C=∩∪∩∪, −C=∪∩∪∩, +D=∩∪∪∩, and −D=∪∩∩∪.

−∘−	$\Gamma_{T}\left[x\left(t\right),T\right]$	$\Gamma_{R}\left[x\left(t\right),T/2\right]$	$\Gamma_{I}\left[x\left(t\right),T/2\right]$	$\Gamma_{G}\left[x\left(t\right),T\right]$
$\Gamma_{T}\left[x\left(t\right),0\right]$	+C	+D	+C	+D
$\Gamma_{G}\left[x\left(t\right),0\right]$	−C	−D	−C	−D
$\Gamma_{R}\left[x\left(t\right),T/2\right]$	−C	+D	−C	+D
$\Gamma_{I}\left[x\left(t\right),T/2\right]$	+C	+D	+C	+D

### Appendix A.4. Cayley Table of Periodic Signal

Here are the detailed calculations for the various elements of Cayley’s table of periodic signals. With the following relations: $\Gamma_{T}\left[x\left(t\right),\tau \right]=x\left(t-\tau \right)$, $\Gamma_{R}\left[x\left(t\right),\tau \right]=x\left(-t+2\tau \right)$, $\Gamma_{I}\left[x\left(t\right),\tau \right]=-x\left(-t+2\tau \right)$, $\Gamma_{G}\left[x\left(t\right),\tau \right]=-x\left(t-\tau \right)$, it comes:

the first raw:
$\Gamma_{T}\left[x\left(t\right),b\right]\;\circ \;\Gamma_{T}\left[x\left(t\right),a\right]=\Gamma_{T}\left[x\left(t-a\right),b\right]=x\left(t-\left(b+a\right)\right)$
$\Gamma_{T}\left[x\left(t\right),b\right]\;\circ \;\Gamma_{R}\left[x\left(t\right),e\right]=\Gamma_{T}\left[x\left(-t+2e\right),b\right]=x\left(-t+2e-b\right)$
$\Gamma_{T}\left[x\left(t\right),b\right]\;\circ \;\Gamma_{I}\left[x\left(t\right),g\right]=\Gamma_{T}\left[-x\left(-t+2g\right),b\right]=-x\left(-t+2g-b\right)$
$\Gamma_{T}\left[x\left(t\right),b\right]\;\circ \;\Gamma_{G}\left[x\left(t\right),c\right]=\Gamma_{T}\left[-x\left(t-c\right),b\right]=-x\left(t-\left(c+b\right)\right)$
for the second raw:
$\Gamma_{R}\left[x\left(t\right),f\right]\;\circ \;\Gamma_{T}\left[x\left(t\right),a\right]=\Gamma_{R}\left[x\left(t-a\right),f\right]=x\left(-t+a+2f\right)$
$\Gamma_{R}\left[x\left(t\right),f\right]\;\circ \;\Gamma_{R}\left[x\left(t\right),e\right]=\Gamma_{R}\left[x\left(-t+2e\right),f\right]=x\left(t-2e+2f\right)$
$\Gamma_{R}\left[x\left(t\right),f\right]\;\circ \;\Gamma_{I}\left[x\left(t\right),g\right]=\Gamma_{R}\left[-x\left(-t+2g\right),f\right]=-x\left(t-2g+2f\right)$
$\Gamma_{R}\left[x\left(t\right),f\right]\;\circ \;\Gamma_{G}\left[x\left(t\right),c\right]=\Gamma_{R}\left[-x\left(t-c\right),f\right]=-x\left(-t+c+2f\right)$
for the third raw:
$\Gamma_{I}\left[x\left(t\right),h\right]\;\circ \;\Gamma_{T}\left[x\left(t\right),a\right]=\Gamma_{I}\left[x\left(t-a\right),h\right]=-x\left(-t+a+2h\right)$
$\Gamma_{I}\left[x\left(t\right),h\right]\;\circ \;\Gamma_{R}\left[x\left(t\right),e\right]=\Gamma_{I}\left[x\left(-t+2e\right),h\right]=-x\left(t-2e+2h\right)$
$\Gamma_{I}\left[x\left(t\right),h\right]\;\circ \;\Gamma_{I}\left[x\left(t\right),g\right]=\Gamma_{I}\left[-x\left(-t+2g\right),h\right]=x\left(t-2g+2h\right)$
$\Gamma_{I}\left[x\left(t\right),h\right]\;\circ \;\Gamma_{G}\left[x\left(t\right),c\right]=\Gamma_{I}\left[-x\left(t-c\right),h\right]=x\left(-t+c+2h\right)$
for the fourth raw:
$\Gamma_{G}\left[x\left(t\right),d\right]\;\circ \;\Gamma_{T}\left[x\left(t\right),a\right]=\Gamma_{G}\left[x\left(t-a\right),d\right]=-x\left(t-a-d\right)$
$\Gamma_{G}\left[x\left(t\right),d\right]\;\circ \;\Gamma_{R}\left[x\left(t\right),e\right]=\Gamma_{G}\left[x\left(-t+2e\right),d\right]=-x\left(-t+2e-d\right)$
$\Gamma_{G}\left[x\left(t\right),d\right]\;\circ \;\Gamma_{I}\left[x\left(t\right),g\right]=\Gamma_{G}\left[-x\left(-t+2g\right),d\right]=x\left(-t+2g-2d\right)$
$\Gamma_{G}\left[x\left(t\right),d\right]\;\circ \;\Gamma_{G}\left[x\left(t\right),c\right]=\Gamma_{G}\left[-x\left(t-c\right),d\right]=x\left(t-c-d\right)$
